# Single nucleotide polymorphism-based visual identification of *Rhodiola crenulata* using the loop-mediated isothermal amplification technique

**DOI:** 10.3389/fpls.2024.1492083

**Published:** 2025-01-16

**Authors:** Li Hao, Xin Shi, Shiyu Wen, Caiye Yang, Yaqi Chen, Samo Yue, Jiaqiang Chen, Kexin Luo, Bingliang Liu, Yanxia Sun, Yi Zhang

**Affiliations:** ^1^ College of Food and Biological Engineering, Chengdu University, Chengdu, China; ^2^ Chengdu Institute of Biology, Chinese Academy of Sciences, Chengdu, China

**Keywords:** medicinal herb, original plant identification, molecular authentication method, 21 naked-eye detection, ITS sequences, using only visual inspection enhance the reaction rate

## Abstract

**Introduction:**

*Rhodiola crenulata* (Hook.f. & Thomson) H.Ohba, a member of the Crassulaceae family, is a traditional Chinese medicine recognized as the original source of Rhodiolae Crenulatae Radix et Rhizoma in the 2020 edition of the China Pharmacopoeia. It has been widely used in both Asia and Europe to enhance stress resistance and reduce fatigue. However, the classification of *Rhodiola* species can lead to confusion, raising safety concerns in the herbal medicine market.

**Methods:**

The cleaved amplified polymorphic sequence (CAPS) RT-PCR was used to identify the single nucleotide polymorphism (SNP) sites, based on which the loop-mediated isothermal amplification (LAMP) was employed to develop the method in *Rh. crenulata* identification. The specific loop backward primers, reaction temperature, reaction time, and color indicators were screened and optimized.

**Results:**

Single nucleotide polymorphism (SNP) sites were identified between Rh. crenulata and two closely related species. Based on the identified SNP sites, the optimal real-time fluorescence LAMP system to identify *Rh. crenulata* was constructed with the most efficient specific loop backward primers, reaction temperature. The final detection system exhibited a sensitivity of up to 1,000 copies of the target DNA, maintaining a constant reaction temperature of 62°C within 35 minutes. To facilitate visualization, we incorporated two color indicators, hydroxy naphthol blue (HNB) and neutral red (N-red), into the reaction system.

**Discussion:**

Collectively, we developed a simple, rapid, specific, sensitive, and visible method to distinguish *Rh. crenulata* from other two Rhodiola species and *Rh. crenulata* hybrids. This approach has significant potential for applications in pharmaceutical industry.

## 1 Introduction

*Rhodiola* plants, belonging to the Crassulaceae family, are important medicinal resources known for their dried roots and rhizomes. In China, 73 species of *Rhodiola* are recorded, primarily in high-altitude and cold regions. Among them, *Rhodiola crenulata* (Hook.f. & Thomson) H.Ohba is uniquely recognized as the original source of Rhodiolae Crenulatae Radix et Rhizoma in the Chinese Pharmacopoeia, 2005 to 2020 edition, while several *Rhodiola* species, such as *Rhodiola kirilowii* (Regel) Maxim., play significant roles in traditional medicine and are noted in Xizang monographs (Tao et al., 2019). *Rhodiola* species possess a variety of medicinal properties and have been traditionally used to enhance physical resistance to stress (Fan et al., 2016; Li et al., 2017; Tinsley et al., 2024) and to reduce fatigue (Jiao et al., 2023; Shikov et al., 2014; Yue et al., 2022) across both Asia and Europe, particularly in Xizang (Tao et al., 2019). Additionally, these plants are recognized as tonics or functional food (Recio et al., 2016). The significant demand for *Rhodiola* species has led to resource depletion due to overexploitation. Moreover, the presence of counterfeits and substitutes, including *Rhodiola fastigiata* (Hook.f. & Thomson) S.H.Fu, *Rh. kirilowii*, and other similarly named *Rhodiola* species, has caused confusion in the herbal medicine market (Tao et al., 2019).

The active ingredients and their concentrations vary among different *Rhodiola* species (Panossian et al., 2010). Mixing these medicinal materials can directly affect clinical safety and efficacy (Xin et al., 2015). However, distinguishing between *Rhodiola* species based on morphology and microscopic characteristics is challenging due to similarities in their roots and rhizomes. While liquid chromatography–mass spectrometry (LS-MS) analysis is effective for identifying medicinal plants, it can be time-consuming (Ganzera and Sturm, 2018; Kuwahara et al., 2019). Thus, there is an urgent need for a rapid and accurate method to differentiate *Rh. crenulata* from other *Rhodiola* species.

DNA-based molecular techniques have gained popularity for species identification due to their accuracy and efficiency (Ganie et al., 2015; Grazina et al., 2020). DNA barcoding methods, such as the nuclear ribosomal internal transcribed spacer 2 (ITS2), the large subunit of ribulose-bisphosphate carboxylase (rbcL), maturase K (matK), and psbA-trnH, have received considerable attention (Feng et al., 2015; Intharuksa et al., 2020; Mahadani and Ghosh, 2013; Mishra et al., 2016; Xu et al., 2022). Chen et al. (2010) first proposed ITS2 as a universal DNA barcode for identifying medicinal plants. Typically, distinguishing species within the same genus relies on detecting single nucleotide polymorphism (SNP) sites. In such cases, techniques like polymerase chain reaction (PCR) (Han et al., 2018), restriction fragment length polymorphism (RFLP) (Xu et al., 2022), cleaved amplified polymorphic sequence (CAPS) reverse transcription polymerase chain reaction (RT-PCR) (Hao et al., 2020), and derived CAPS (dCAPS) RT-PCR (Hao et al., 2023) are commonly used. However, these methods can be time-consuming and dependent on thermal cyclers. In contrast, loop-mediated isothermal amplification (LAMP) is a thermal cycler-free method known for its high sensitivity and rapid performance at a constant temperature. LAMP employs a DNA polymerase with strand-displacement activity and two pairs of primers for sequence amplification (Soroka et al., 2021). To enhance the reaction rate and visualize results, loop primer and colorimetric sensors, such as hydroxy naphthol blue (HNB) and neutral red (N-red), can be incorporated (Saejung et al., 2024; Yang et al., 2024). Recently, LAMP has gained traction for SNP detection and genotyping (Ding et al., 2019; Hyman et al., 2022; Sharafdarkolaee et al., 2019), demonstrating great potential for identifying *Rhodiola* species.

In this study, we identified several SNPs in the ITS2 region among various *Rhodiola* species. Based on one of these SNP sites and the LAMP technique, we developed a rapid, convenient, and efficient method for visually distinguishing *Rh. crenulata* from the common substitutes *Rh. kirilowii* and *Rh. fastigiata*, as well as *Rh. crenulata* hybrids, showcasing significant future application prospects.

## 2 Materials and methods

### 2.1 Plant materials

The plant materials for this study included *Rh. crenulata* [voucher specimen: Y.S. Chen et al. 13-0349 (PE)], *Rh. kirilowii* [voucher specimen: FLPH Xizang Expedition 12-0465 (PE)], *Rh. fastigiata* [voucher specimen: PE Xizang Expedition PE6468 (PE)], and a hybrid (*Rh. crenulata* × *Rh. fastigiata*). These species were provided by the Xizang Rhodiola Pharmaceutical Holding Company, Xizang, China, and were identified by professor Yuehua Wang of Chengdu University. Detailed collection information is provided in **Supplementary Table S1**. The 12 batches of *Rhodiola* decoction pieces were purchased from the Lotus Pond Chinese herbal medicine market in Chengdu, Sichuan province, China.

### 2.2 SNP analysis among *Rh. crenulata*, *Rh. Kirilowii*, and *Rh. fastigiata*

A total of 149 ITS2 sequences of *Rhodiola* species, including *Rh. crenulata*, *Rh. kirilowii*, and *Rh. fastigiata*, were downloaded from NCBI (https://www.ncbi.nlm.nih.gov/), with accession numbers listed in **Supplementary Table S2**. The sequences were aligned and trimmed using MEGA 11, and nucleotide diversity (Pi) was calculated using DnaSP6, with a window width and step size set to 1. The Pi values were used to identify potential SNP sites and were visualized using GraphPad Prism 9. The concentrated region (from position 162 bp to 177 bp) containing the potential SNP sites was analyzed for base preference using the R package *ggseqlogo* with default parameters.

Genomic DNA was extracted from the leaves and decoction pieces of the *Rhodiola* species using the Plant Genomic DNA Kit (TIANGEN Biotech Co., Ltd., Beijing, China). The concentration of the extracted DNA was measured using a NanoDrop spectrophotometer (Thermo Fisher Scientific Corporation, USA). The ITS2 fragments were amplified with primers RhITS1-F and RhITS4-R (**Supplementary Table S3**) under standard PCR conditions and sequenced by Sangon Biotech Co., Ltd. (Shanghai, China). The sequencing results were aligned in DNAMAN 7.0 software to confirm the interspecific SNP sites between *Rh. crenulata* and the other two *Rhodiola* species.

### 2.3 CAPS analysis

The ITS2 fragments were amplified from the extracted DNA using primers RhITS1-F and RhITS4-R (**Supplementary Table S3**) in a reaction volume of 20 μL. The PCR products were then purified and digested with *Bgl*I (New England Biolabs, USA). Following a digestion period of 2 h, the products were separated by 2% agarose gel electrophoresis for band detection.

### 2.4 Plasmid construction and template preparation for LAMP reaction

The ITS2 sequences from *Rh. crenulata* and *Rh. fastigiata* were cloned into the pTOPO vector using the CV15-Zero Background pTOPO-TA Simple Cloning Kit (Aidlab Biotechnologies Co., Ltd., Beijing, China). The recombined plasmids were subsequently sequenced and verified by Sangon Biotech Co., Ltd. (Shanghai, China). Following purification with the HighPure Plasmid Mini Kit (Aidlab Biotechnologies Co., Ltd., Beijing, China) and concentration measurement using a NanoDrop spectrophotometer (Thermo Fisher Scientific Corporation, USA), the plasmids were diluted to different copy numbers (from 10^8^ to 10^1^) based on the following formula:


$$\begin{array}{c} \text{Copies/}\mu \text{L}=\\ \frac{\text{Concentration}\left(\frac{\text{ng}}{\text{μL}}\right)\times 6.02\times 10^{23}\left(\text{copies}/\text{mol}\right)\times 10^{-9}\left(\text{g}/\text{ng}\right)}{\text{bases }\left(\text{bp}\right)\times 660\;\left(\text{dalton}/\text{bp}\right)} \end{array}$$

### 2.5 LAMP primer design

The LAMP primers, including F3, B3, F1C-F2 (FIP), B1C-B2 (BIP), and the backward-side loop primer (LB), were designed based on the SNP region in the ITS2 sequence using the online software PrimerExplorer V5 (http://primerexplorer.jp/lampv5e/index.html). The conventional LAMP primer set consisted of two inner primers (FIP and BIP) and two outer primers (F3 and B3). The SNP sites were flanked by the inner primers (B1 and B2) and located in the loop domain of the LAMP products. Specific LB primers (LBRc1/2/3 for *Rh. crenulata* and LBRh1/2/3 for the other two species) were designed based on the first SNP-concentrated region. LBRc1/2 and LBRh1/2 differed in the number of SNPs but were similar in length [11 nucleotides (nt)] and had SNPs located at the 5′ and 3′ ends, as well as in the middle of the primers. LBRc3 and LBRh3, each 17 nt long, contained four SNP sites distributed at the 5′ end and the middle of the primers. All primers are listed in **Supplementary Table S3**.

### 2.6 Real-time fluorescence LAMP reaction

The total volume of the real-time fluorescence LAMP reaction was 25 μL, which included the following components: 10 × Isothermal Amplification Buffer, 8 mM MgSO_4_, 1.4 mM dNTPs, 1.6 μM of each FIP and BIP primer, 0.2 μM of each F3 and B3 primer, 0.4 μM LB primer, 8 U *Bst* 2.0 WarmStart DNA polymerase (New England Biolabs, USA), 0.5 μL of 50 × LAMP fluorescence dye (New England Biolabs, USA), and 1 μL of plasmid template (10^4^ copies). The amplification reaction was performed at a constant temperature for 90 min using the QuantStudio 3 Real-Time PCR System (Applied Biosystems, California, USA). All real-time fluorescence LAMP reactions were performed with three biological replicates, each consisting of three technical replicates.

### 2.7 The sensitivity and specificity analysis of the LAMP method

The sensitivity of the LAMP reaction was evaluated using a 10-fold serial dilution of the plasmid template, ranging from 10^8^ to 10^1^ copies. The specificity of LBRh3 was assessed by mixing plasmid templates with the same total copy number (10^6^) but varying the ratios of RfITS2 to RcITS2 (100%, 10%, 1%, 0.1%, and 0%).

### 2.8 Visualization of LAMP detection

HNB and N-red were used as colorimetric indicators for visualized LAMP detection. In the reaction system, the 50 × LAMP fluorescence dye was replaced with either 0.12 mM HNB or 0.1 mM N-red, along with 1 μL of plasmid template (10^4^ copies) or 0.5 μL of DNA (20 ng/μL) extracted from *Rhodiola* species. The remaining components were consistent with those in the real-time fluorescence LAMP reaction system described previously. The reaction was conducted at 62°C for 35 min using a VeritiPro 96-Well Thermal Cycler (Applied Biosystems, California, USA). The final visualized results were captured using a Sony α6000 camera (Sony, Japan) against a white background. All visualized LAMP reactions were performed in triplicate.

### 2.9 Statistical analysis

The real-time fluorescence signals of the LAMP reactions were collected using the QuantStudio 3 Real-Time PCR System and visualized with GraphPad Prism 9 or OriginPro 2022. The cycle quantification (Cq) value was defined as the detection time. All experiments were conducted in triplicate. Statistical analyses were performed using Duncan’s multiple range test via one-way ANOVA or two-tailed Student’s *t*-test in SPSS 25.

## 3 Results

### 3.1 SNP identification between *Rh. crenulata* and the other two *Rhodiola* species

**Figure 1:**
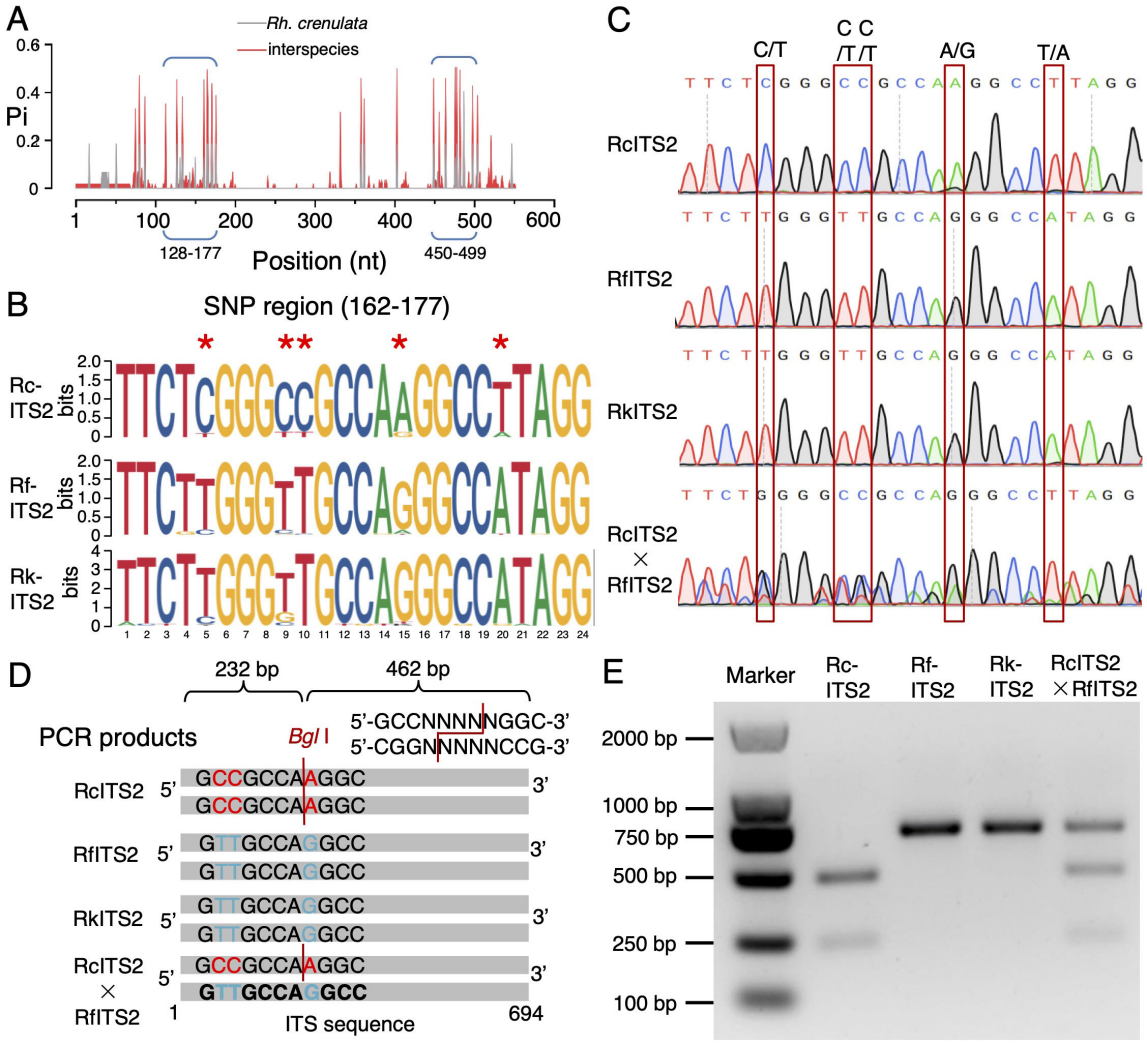
*SNP analysis and identification of Rh. crenulata* and two other *Rhodiola* species. **(A)** Nucleotide diversity (Pi) of the ITS2 region in *Rh. crenulata* and two other *Rhodiola* species. A high Pi value indicated significant hypervariability in nucleotides, either intra- or interspecifically. SNP-concentrated regions are marked with blue double bracket. **(B)** Base preference analysis of the first SNP-concentrated region (162 to 177 bp). Potential SNP sites are highlighted with red stars. **(C)** Sequencing peak map of PCR products from four *Rhodiola* species. The SNP site is indicated by a red box. **(D)** Schematic diagram illustrating the *Bgl*I restriction site in the ITS2 region across *Rhodiola* species. **(E)** CAPS analysis of PCR products from the four *Rhodiola* species. Products from *Rh. crenulata* were cleaved into two fragments (232 and 462 bp), while those from *Rh. crenulata* hybrid (*Rh. crenulata* × *Rh. fastigiata*) were cleaved into three fragments (232, 462, and 694 bp). The PCR products from *Rh. fastigiata* and *Rh. kirilowii* remained uncleaved. Marker, DL2000 DNA ladder.

To differentiate *Rh. crenulata* from its counterfeits and substitutes, we focused on the ITS2 gene to identify potential SNPs. We obtained a total of 149 sequences of three *Rhodiola* species from GenBank, including *Rh. crenulata*, *Rh. fastigiata*, and *Rh. kirilowii* (**Supplementary Table S2**). Nucleotide diversity (Pi) was used to access interspecific SNPs between *Rh. crenulata* and the other species. We identified multiple sites with higher interspecific Pi values (>0.37) and lower intraspecific Pi values (<0.19) (**Figure 1A**; **Supplementary Table S4**), which were considered candidate SNPs. These sites were concentrated in two fragments, each 50 nt long (**Figure 1A**).

To further verify the differentiation among the *Rhodiola* species, we conducted a base preference analysis. This revealed five specific nucleotide preferences in *Rh. crenulata* within the first SNP-concentrated region (position 162 bp to 177 bp): C162, C166, C167, A172, and T177. In contrast, *Rh. fastigiata* and *Rh. kirilowii* were more likely to have T162, T166, T167, G172, and A177 at these positions (**Figure 1B**). A more dispersed nucleotide divergence was observed between *Rh. crenulata* and the other two *Rhodiola* species in the second SNP-concentrated region (**Supplementary Figure S1A**). Consequently, the five SNP sites in the first region were considered critical for distinguishing *Rh. crenulata.*

To determine whether these SNP sites were homozygous, we amplified ITS2 fragments from the DNA of each *Rhodiola* species and sequenced the PCR products. All sites in the first SNP-concentrated region exhibited single peaks, confirming their homozygosity in *Rh. crenulata*, *Rh. fastigiata*, and *Rh. kirilowii* (**Figure 1C**; **Supplementary Figure S1B**). However, double peaks were observed at the SNP positions in the *Rh. crenulata* hybrid (**Figure 1C**). Notably, a key restriction enzyme site (*Bgl*I) was formed by the specific bases C166 and C167 in *Rh. crenulata* (**Figure 1D**). CAPS analysis demonstrated that the PCR products amplified from *Rh. crenulata* were cleaved into two fragments (462 and 232 bp) by *Bgl*I, while those from *Rh. fastigiata* and *Rh. kirilowii* were not cleaved (**Figure 1E**). Moreover, we amplified the same 694-bp ITS2 fragment from the *Rh. crenulata* hybrid (*Rh. crenulata* × *Rh. fastigiata*) (**Supplementary Figure S1C**), which resulted in three fragments (232 bp, 462 bp, and 694 bp) following digestion with *Bgl*I (**Figure 1E**). These results indicated that positions 166 and 167 were key SNP sites for effectively distinguishing *Rh. crenulata* from the other two *Rhodiola* species and its hybrid.

### 3.2 Application design of the LAMP technique in *Rh. crenulata* identification

**Figure 2:**
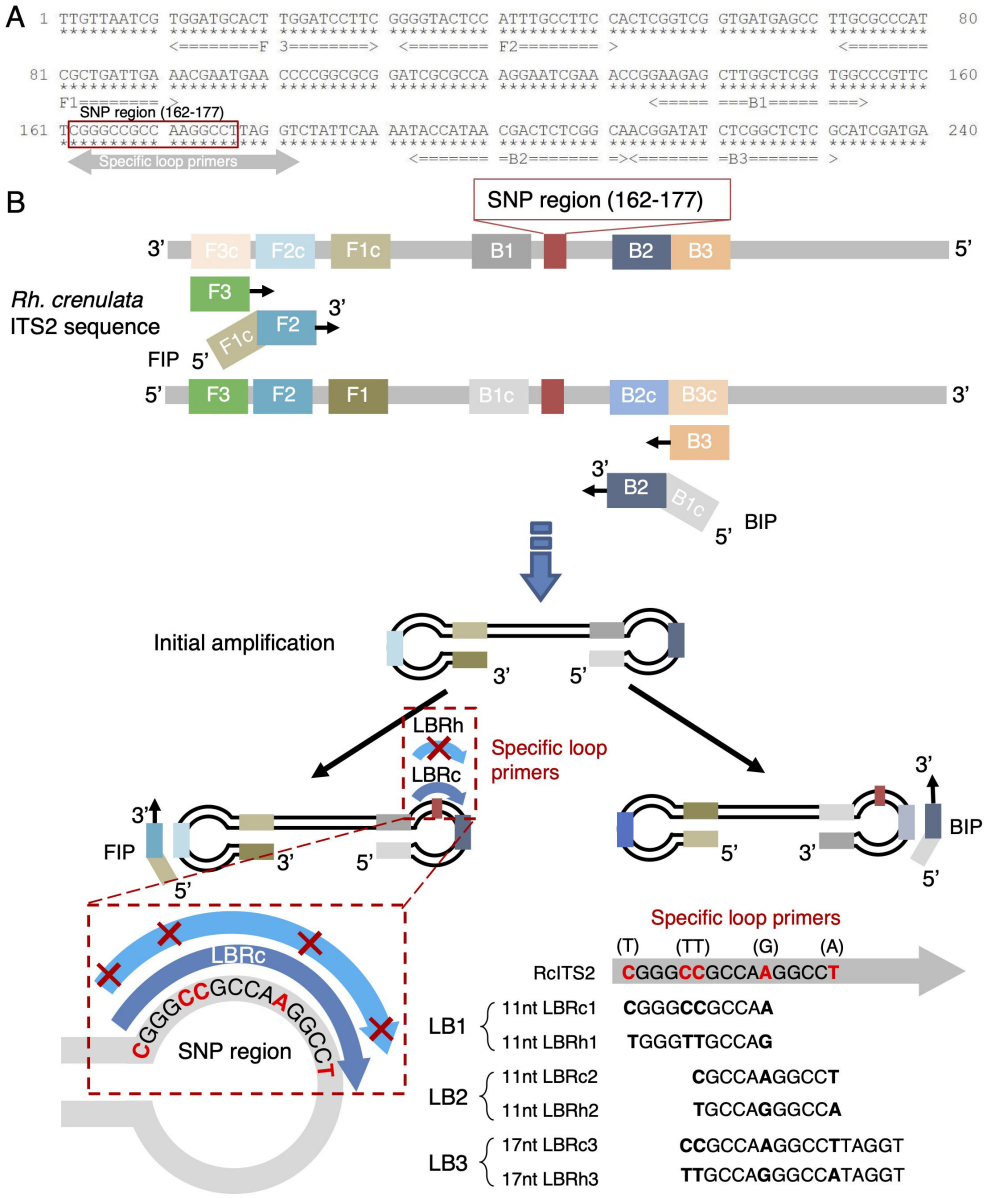
Schematic diagram of LAMP detection of *Rhodiola* species. **(A)** Location of LAMP primers within the ITS2 region of *Rh. crenulata*. The red box indicates the SNP region, while the double-headed gray arrow shows the positions of the specific loop primers. The stars under the nucleotides are for position clarification. **(B)** Overview of LAMP detection for *Rhodiola* species. The SNP region is highlighted in red, with bolded SNP sites shown in the specific loop primers. The symbol “✕” in LB-Rh represents non-complementary paring sites.

To rapidly distinguish *Rh. crenulata* from the other two *Rhodiola* species, we employed the LAMP technique, which eliminated the need for thermocycling equipment and simplified the procedures. The primers and strategy for differentiating *Rh. crenulata* are illustrated in **Figure 2**. Conventional LAMP primers (FIP, BIP, F3, and B3) were designed to amplify the ITS2 fragments in *Rhodiola* species, while specific LB primers targeting the first SNP-concentrated region were used to enhance the LAMP reaction. The design and binding characteristics of the LB primers significantly influence the amplification efficiency. The LBRc primers (LBRc1/2/3) were perfectly complementary to the SNP region of *Rh. crenulata* but exhibited mismatches at the 5′ and 3′ ends, as well as in the middle regions, preventing effective binding to the SNP regions of the other two *Rhodiola* species (**Figure 2B**). Consequently, *Rh. crenulata* was expected to be detected earlier than the other two species using the specific LBRc primer. Similarly, the specific LBRh primer facilitated the earlier detection of *Rh. fastigiata* and *Rh. kirilowii*. This allowed for the identification of optimal reaction times to distinguish different *Rhodiola* species within the LAMP system. To enhance detection visibility, colorimetric indicators such as HNB and N-red were incorporated into the LAMP reaction.

### 3.3 Optimization of the LAMP reactions

**Figure 3:**
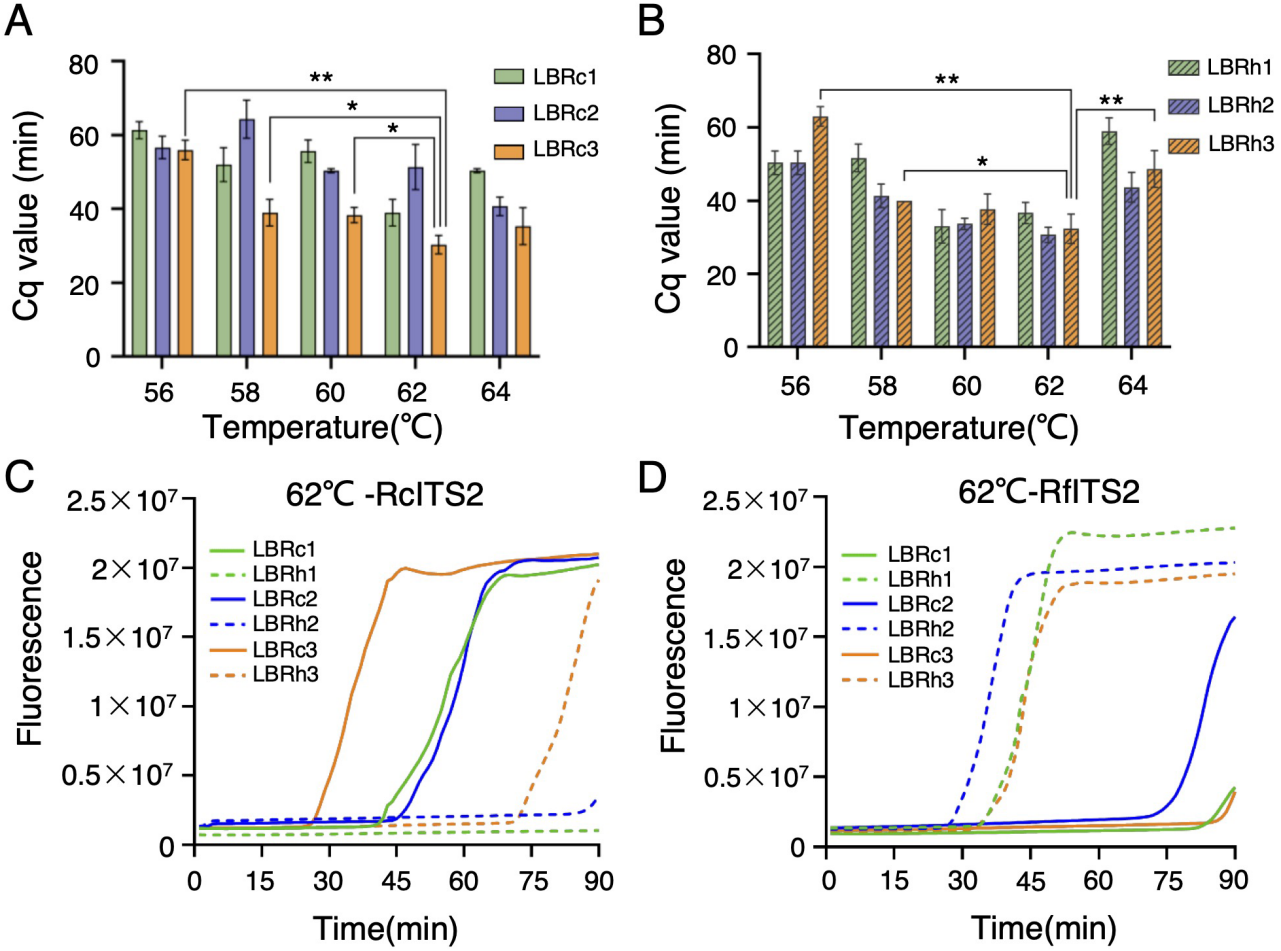
Construction of the LAMP reaction system for *Rhodiola* species identification. **(A)** Detection time (Cq value) of RcITS2 using LB-Rc primers at different temperatures in a real-time fluorescence LAMP reaction. **(B)** Detection time (Cq value) of RfITS2 using LB-Rh primers at varying temperatures in a real-time fluorescence LAMP reaction. Statistical analyses were performed using Duncan’s multiple range test within a one-way ANOVA framework (*p < 0.05, **p < 0.01). Error bars indicate means ± standard deviation (SD) from three independent replicates. **(C, D)** Discrimination plot from the real-time fluorescence LAMP reaction using RcITS2 **(C)** or RfITS2 **(D)** plasmids as templates at 62°C. The plot was the representative of three independent experiments.

To optimize the LAMP reaction conditions for identifying *Rh. crenulata*, we constructed two plasmids containing the ITS2 sequences from *Rh. crenulata* and *Rh. fastigiata*, which served as reaction templates. We then conducted a series of real-time fluorescence LAMP reactions using specific primers at temperatures ranging from 56°C to 64°C in 2°C intervals to verify the most effective LB primer and reaction temperature for identifying *Rhodiola* species. The Cq value was used to indicate the detection time of the target species. Results revealed that the mean Cq value for the LBRc3 primer was consistently lower than that of LBRc1/2 across all reaction temperatures, particularly at 62°C, where its Cq value was significantly lower than at other temperatures (**Figure 3A**; **Supplementary Table S5**). Similarly, LBRh3 exhibited a lower mean Cq value at 62°C, with significant differences compared to other temperatures (56°C, 58°C, and 64°C), except at 60°C (**Figure 3B**; **Supplementary Table S5**). These findings indicated that the 17-nt LBRc3/Rh3 primers, which contained four SNP sites, demonstrated a high amplification rate at 62°C, identifying it as the optimal reaction temperature for the LAMP system.

We validated the influence of the reaction template on amplification rates. When amplifying RcITS2, the amplification rates for the three LBRc primers were significantly higher than those for the LBRh primers, which contained mismatches, across the temperature range of 56°C to 64°C (**Figures 3C**; **Supplementary Figure S2**). Conversely, using a plasmid containing RfITS2 as the template resulted in lower amplification efficiency for the LBRc primers compared to the LBRh primers at all temperature gradients (**Figures 3D**; **Supplementary Figure S2**).

**Figure 4:**
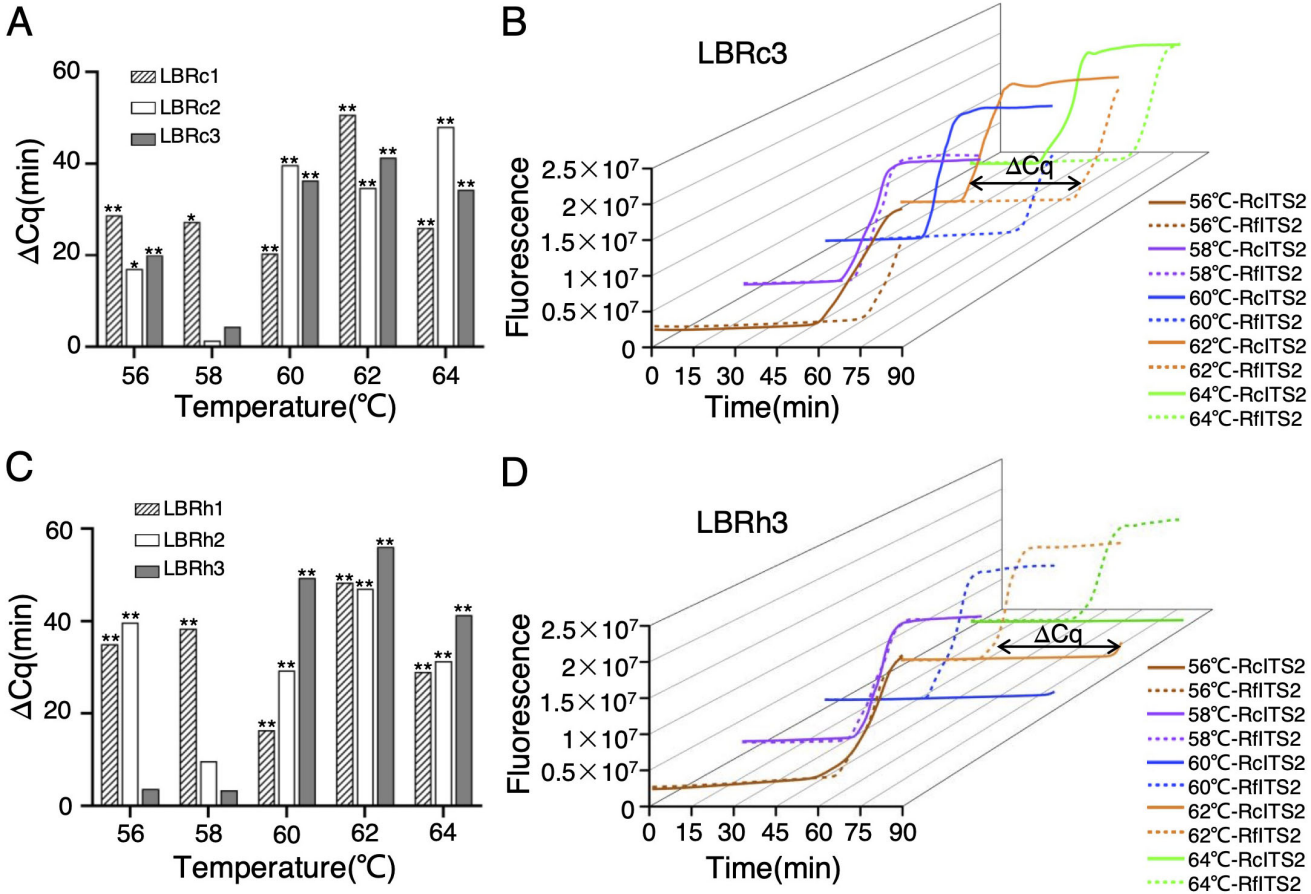
Time gaps between the detection of *Rh. crenulata* and *Rh. fastigiata* using specific LB primers. **(A, C)** Time gaps (ΔCq) between RcITS2 and RfITS2 detection using LBRc primers **(A)** and LBRh primers **(C)** at different temperatures. **(B, D)** Three-dimensional (3D) view of the time gap (ΔCq) between RcITS2 and RfITS2 detection using LBRc3 **(B)** and LBRh3 **(D)** at varying temperatures. All experiments were conducted in triplicate. Significance analysis was determined using a two-tailed Student’s *t*-test in SPSS 25 (*p < 0.05, **p < 0.01).

In particular, except for LBRc2, the maximum differences in Cq values across different templates were observed at 62°C (**Figures 4**; **Supplementary Figures S3**, **S4**). The time gap (ΔCq) exceeded 40 min when detecting RcITS2 and RfITS2 using LBRc1 or LBRc3 at this temperature (**Figures 4A**; **Supplementary Figure S3A**), which was particularly evident in 3D representations (**Figures 4B**; **Supplementary Figures S4A**, **S4B**). When LBRh3 was used, the time gap exceeded 55 min at 62°C (**Figures 4C, D**; **Supplementary Figure S3B**). Overall, both LBRc3 and LBRh3 effectively identified *Rh. crenulata*, *Rh. fastigiata*, and *Rh. kirilowii* in the LAMP system at 62°C.

### 3.4 Sensitivity and specificity analysis of the LAMP system

Although the LAMP reaction with LBRc3 or LBRh3 at 62°C effectively distinguished between the RcITS2 and RfITS2 plasmid (at 10^4^ copies), the presence of considerable impurities in the samples highlighted the need for a LAMP method with high sensitivity and specificity for practical detection.

**Figure 5:**
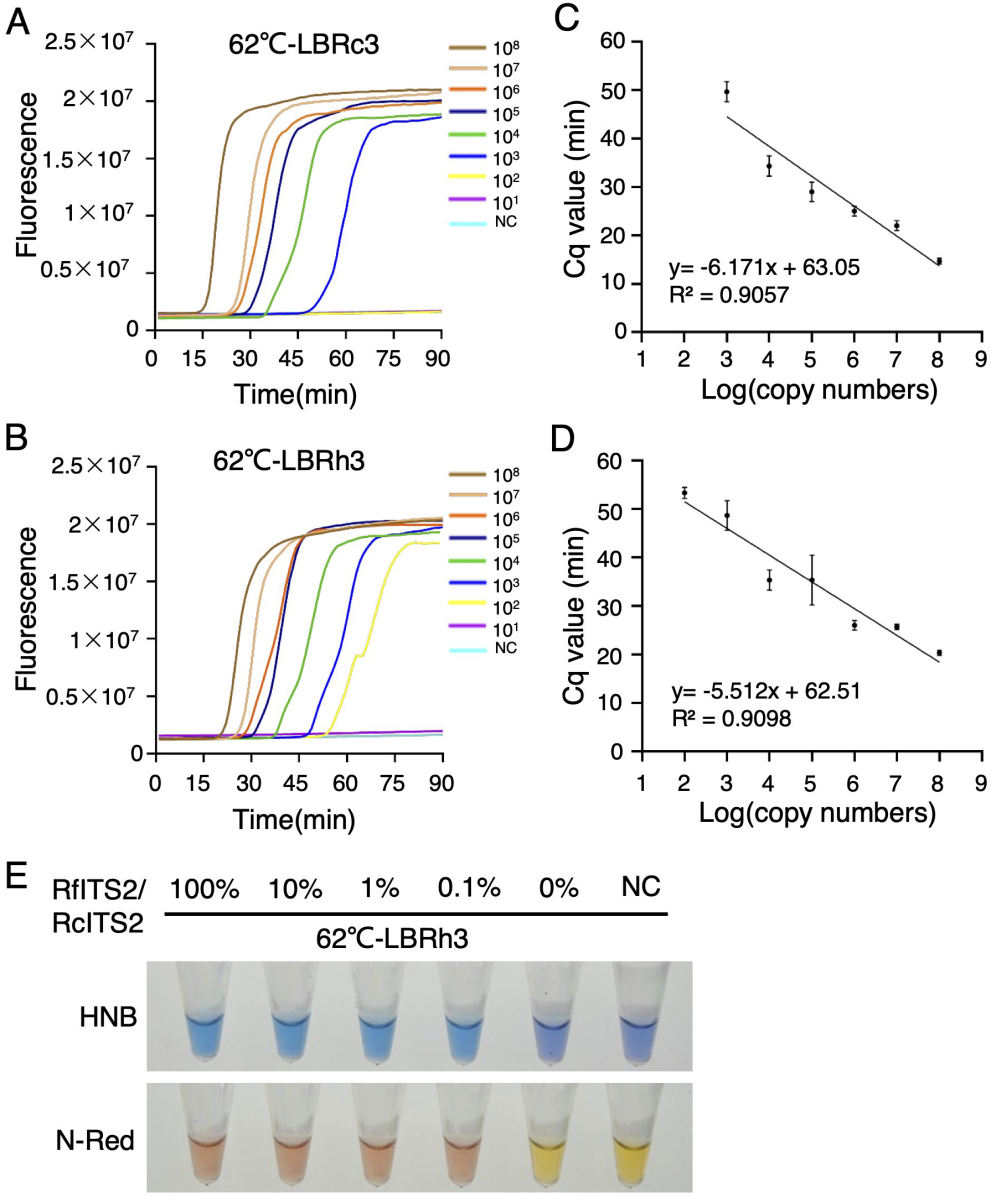
Sensitivity and specificity of the LAMP system. **(A, B)** Real-time fluorescence LAMP detection with different copy numbers of plasmid templates using LBRc3 **(A)** and LBRh3 **(B)** at 62°C. **(C, D)** Linear correlation between detection time (Cq value) and template copy number for LBRc3 **(C)** and LBRh3 **(D)**. Error bars represent mean ± standard deviation (SD) from three independent biological replicates. **(E)** Specificity analysis of LBRh3 in detecting RfITS2 using colorimetric indicators in the LAMP system. All experiments were repeated three times.

In the medicinal materials of *Rh. crenulata*, it is common for adulterants to be mixed in. Consequently, we focused on evaluating the sensitivity of the LBRh3 primer to effectively distinguish these adulterants from *Rh. crenulata*. To assess this sensitivity, we utilized plasmid templates with copy number gradients ranging from 10^8^ to 10^1^. Real-time LAMP reaction results showed that the lowest copy number of the RcITS2 plasmid, efficiently amplified by LBRc3, was 10^3^, while the lowest copy number of the RfITS2 plasmid, amplified by LBRh3, was 10^2^ at 62°C (**Figures 5A, B**). The LAMP system demonstrated sufficient sensitivity to handle extracted genomic DNA samples with concentrations between 10^4^ and 10^5^ copies. Additionally, a linear correlation was established between the Cq value and plasmid concentration (**Figures 5C, D**), indicating that template concentration could be estimated based on the Cq value.

In the herbal medicine market, *Rh. crenulata* is often adulterated with *Rh. fastigiata* and *Rh. kirilowii*, raising safety and quality concerns. Thus, the specificity and sensitivity of LBRh3 in identifying these *Rhodiola* species, particularly *Rh. fastigiata* and *Rh. kirilowii*, is crucial. To enhance the practicality and applicability of the LAMP detection system, we replaced fluorescence dyes with colorimetric indicators such as HNB and N-red. The color of the reaction system changed when the target gene was efficiently amplified. We generated a set of mixed plasmid templates with identical copy numbers (10^6^) but varying RfITS2/RcITS2 ratios (100%, 10%, 1%, 0.1%, and 0%). Notably, a significant color change was observed by the naked eye in all experimental groups with RfITS2/RcITS2 ratios ranging from 100% to 0.1%, except in the group without the RfITS2 plasmid (**Figure 5E**).

### 3.5 Application of the LAMP system in *Rhodiola* species identification

**Figure 6:**
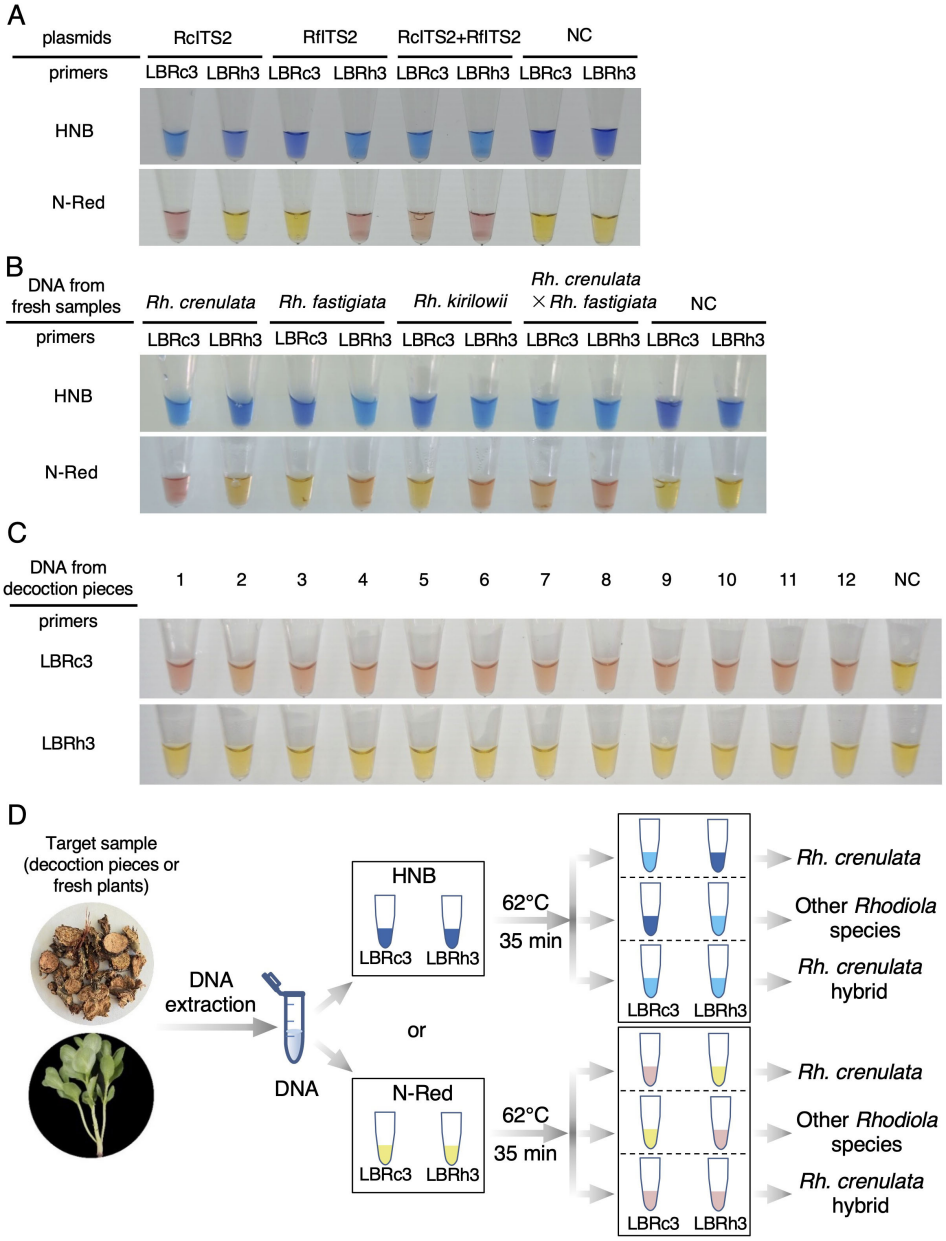
Visualized detection of *Rh. crenulata* using the LAMP system. **(A)** LAMP detection using HNB and N-red with plasmid templates. **(B)** Application of visual detection in DNA samples from four *Rhodiola* species. NC, negative control. Each experiment was repeated three times. **(C)** Application of the visual LAMP system with N-red in identifying *Rhodiola* decoction pieces. NC, negative control. Each experiment was repeated three times. **(D)** Flowchart illustrating the process of identifying *Rh. crenulata* using the visual LAMP system.

We first validated the visualized detection of the LAMP reaction using plasmid templates. Based on the Cq values obtained for LBRc3/Rh3 in the amplification of RcITS2/RfITS2 at 62°C (**Figures 3C, D**, **4B, D**), we set the reaction time for visualized detection to 35 min. Compared to the control group without plasmid templates, the color of the reaction system changed to light blue (with HNB) or pink (N-red) after 35 min for the following samples: RcITS2 plasmid + LBRc primer, RfITS2 plasmid + LBRh primer, RcITS2 plasmid + RfITS2 plasmid +LBRc primer, and RcITS2 plasmid + RfITS2 plasmid + LBRh primer (**Figure 6A**). These color changes were clearly observable to the naked eye and were consistent across both colorimetric indicators (**Figure 6A**).

To test the LAMP detection system for identifying *Rhodiola* species, we used DNA extracted from leaves of four *Rhodiola* species as templates in the LAMP reaction. After 35 min at 62°C, a color change was observed in the reaction tubes containing DNA templates from *Rh. crenulata* or *Rh. crenulata* hybrids when LBRc3 was used as the primer (**Figure 6B**). Conversely, when LBRh3 served as the primer, the reaction tubes with DNA templates from *Rh. fastigiata*, *Rh. kirilowii*, or *Rh. crenulata* hybrids also exhibited a color change (**Figure 6B**).

To further verify the LAMP detection system for decoction piece identification, we performed reactions using DNA extracted from 12 batches of market samples with the LBRc3 and LBRh3 primers. After a 35-min reaction at 62°C, a color change was noted in the reaction tubes with LBRc3 primer, which turned light blue or pink. In contrast, those with the LBRh3 primer showed no color change, remaining blue or yellow (**Figure 6C**; **Supplementary Figure S5**). Based on these results, all the market samples were identified as *Rh. crenulata.*

In summary, the identification of target *Rhodiola* species requires simultaneous LAMP reactions using either the LBRc3 or LBRh3 primer, with two available calorimetric dyes (HNB and N-red). Three distinct color change scenarios can be observed in the reaction tubes. If the tubes with the LBRc3 primer show light blue (with HNB) or pink (N-red) while the LBRh3 tubes remain blue or yellow, the target species is identified as *Rh. crenulata*. If the LBRc3 tubes remain invariant in color (blue or yellow) while those with LBRh3 change to light blue or pink, the detected species is identified as another *Rhodiola* species, excluding *Rh. crenulata*. If both reaction tubes exhibit color changes, the target species is considered a hybrid of *Rh. crenulata* (**Figure 6D**).

Taken together, we have developed a rapid and convenient method using the LAMP system to distinguish *Rh. crenulata* within 35 min, with results that were visibly identifiable to the naked eye. This method has been validated with various materials including fresh samples of *Rh. crenulata*, *Rh. fastigiata*, *Rh. kirilowii*, and *Rh. crenulata* hybrids, and decoction pieces of *Rh. crenulata*.

## 4 Discussion

*Rhodiola* species have a long history as medicinal plants in Asia and Europe. Among them, *Rh. crenulata* and *Rh. kirilowii* are documented in various Chinese monographs, but only *Rh. crenulata* is included in the Chinese Pharmacopoeia 2020. Because of its significant clinical efficacy and high commercial value, *Rh. crenulata* is classified as a rare and endangered plant in China, primarily attributed to exploitation. Furthermore, the frequent mixing of different *Rhodiola* species in the herbal medicine market raises concerns regarding efficacy and safety. The pharmaceutical compositions and their contents vary among *Rhodiola* species, which can pose risks to food and medicine safety. Therefore, the rapid and accurate identification method developed in this study is crucial for enhancing market supervision within the pharmaceutical industry and ensuring the proper use of *Rh. crenulata*.

The ITS2 region is an effective DNA barcode for species identification, including for *Rhodiola rosea* L. and *Rh. crenulata* (Zhu et al., 2017). SNPs have emerged as efficient tools for identifying both intraspecific and interspecific variations in medicinal plants (Chen et al., 2024; Guo et al., 2017; Xu et al., 2021). A previous study identified 35 variable sites in 32 ITS2 sequences from 10 *Rhodiola* species (Xin et al., 2015). In this study, we identified two 50-nt regions within the ITS2 that contain multiple SNP sites among three *Rhodiola* species (**Figure 1A**), demonstrating sufficient interspecific divergence to serve as potential DNA markers. Reliable and stable SNP sites are essential for unambiguous differentiation between species (Mishra et al., 2016). Various technologies exist for SNP detection, including DNA sequencing, PCR, and PCR-derived methods. We employed two methods to validate the SNP sites between *Rh. crenulata* and the other two *Rhodiola* species. The first method, known as CAPS, involved traditional molecular approaches using the restriction endonuclease *Bgl*I (**Figure 1E**). However, this process is time-consuming, requiring multiple steps such as DNA extraction, PCR amplification, enzyme digestion, and gel electrophoresis, which are dependent on thermocyclers.

The second technique we utilized was the LAMP method, a highly sensitive and cost-effective technology that leverages primer mismatches and differentiated amplification rates. The hybridization reaction between the loop primer and the target sequence is significantly influenced by the characteristics of mismatched bases—especially in their number, type, and location. Generally, mismatches involving C–G pairs have a more pronounced impact on the reaction compared to A–T pairs. Regardless of the mismatch type, probe primers with a mismatch at the center demonstrate lower amplification efficiency than those with mismatches at the 5′ or 3′ ends (Ding et al., 2019; Duan et al., 2010). Specifically, at the probe center, T → A transitions exert a stronger effect on signal intensity compared to C → T transitions (Duan et al., 2010). Likewise, the LBRc3/Rh3 primers, which contained a C → T mismatch at the 5′ end and a T → A mismatch at the center, exhibited faster amplification rates than the LBRc1/Rh1 primers, which had two mismatches at the center (**Figures 2B**, **3A, B**).

Since the initial identification of medicinal plants using DNA barcoding-targeted LAMP techniques in 2007 (Sasaki and Nagumo, 2007), this approach has been successfully applied to identify several species, including *Hedyotis diffusa* Willd (Li et al., 2013), *Taraxacum formosanum* Kitam (Lai et al., 2015), *Crocus sativus* L (Zhao et al., 2016), *Dendrobium officinale* Kimura & Migo (Yang et al., 2019), and *Portulaca oleracea* L (Xu et al., 2023). The high sensitivity and specificity of the LAMP technique are particularly important for detecting low-quality DNA samples, which may be extracted using simple methods or from mixed DNA sources. In our study, the species-specific primers LBRc3 and LBRh3 amplified as few as 10^3^ copies of RcITS2 and 10^2^ copies of RfITS2, respectively (**Figures 5A, C**), demonstrating performance comparable to previously reported methods (Ding et al., 2019) and high-resolution melting (HRM) analysis (Ren et al., 2017). This sensitivity helps to mitigate false negatives that may occur during practical applications. Furthermore, LBRh3 was able to successfully distinguish a 0.1% RfITS2 plasmid (approximately 10^3^ copies) from a 99.9% RcITS2 plasmid (**Figure 5E**), indicating that the LAMP detection system developed in this study is effective for identifying mixed samples. To confirm the broad applicability of our LAMP system, we obtained ITS2 sequences of various *Rhodiola* species from GenBank, including *Rhodiola yunnanensis* (Franch.) S.H.Fu, *Rhodiola tangutica* (Maxim.) S.H.Fu, *Rhodiola sacra* (Prain ex Raym.-Hamet) S.H.Fu, *Rh. rosea*, and eight other *Rhodiola* species from GenBank. Base preference analysis illustrated that the ITS2 sequences from these species shared similar sequences in the SNP region (162–177 bp) with *Rh. kirilowii* and *Rh. fastigiata*, except for *Rh. crenulata* (**Supplementary Figure S6**). Therefore, our LAMP system can theoretically distinguish *Rh. crenulata* from the analyzed *Rhodiola* species.

In addition to sensitivity and specificity, the time required for identification methods is a significant consideration. LAMP is a rapid and convenient technique widely used in pathogen detection (Shen et al., 2020; Yang et al., 2018), disease diagnosis (Besuschio et al., 2017; Gawande et al., 2022), and herbal medicine identification (Xu et al., 2023; Yang et al., 2019). Most LAMP reactions typically require approximately 40 min. In this study, we successfully identified *Rh. crenulata* using the LAMP system in just 35 min, significantly shorter than the 2-h duration needed for traditional PCR. However, the complete identification process, including DNA extraction, took about 1.5 h. High-quality genomic DNA is essential for ensuring an efficient LAMP reaction. Common DNA extraction methods, such as the CTAB method and extraction kits, are often time-consuming. Thus, there is a pressing need to develop a rapid DNA extraction method.

Moreover, we improved the LAMP system by incorporating pre-added colorimetric indicators, which provided visible detection and significantly reduced costs. This visualized LAMP system offers a reliable and scalable technology for identifying medicinal plants compared to the CAPS method (**Figure 1E**), providing advantages in speed and convenience. In the future, combining the LAMP technique with DNA barcoding holds great promise for accurately identifying medicinal materials and resolving species confusion in the herbal medicine market.

As a rapid and sensitive molecular identification technology, LAMP amplification produces high concentrations of products, which increases the risk of false positives due to aerosol contamination. It is essential to design multiple primers based on SNP region analysis to minimize false positives. Additionally, traditional CAPS analysis can serve as a supplementary verification method by digesting products with specific restriction enzymes. However, the sensitivity of LAMP technology may limit its application, as low concentrations of adulterants in samples could remain undetected, leading to false negatives. To enhance the accuracy, it is advisable to combine LAMP with more precise identification methods.

## 5 Conclusion

In this study, we developed a rapid, convenient, sensitive, and visual method for identifying *Rh. crenulata* in just 35 min using SNP sites and the LAMP system, showcasing significant potential for application in the herbal medicine industry.

## Data Availability

The datasets presented in this study can be found in online repositories. The names of the repository/repositories and accession number(s) can be found in the article/**Supplementary Material**.
