# Supplementary material for: Single nucleotide polymorphism-based visual identification of Rhodiola crenulata using the loop-mediated isothermal amplification technique
Source: Front Plant Sci. 2025 Jan 16;15:1492083. doi: 10.3389/fpls.2024.1492083 (PMC11779703; doi:10.3389/fpls.2024.1492083)
Supplement: Supplementary file 1 [file DataSheet1.pdf]

Supplementary figures

Single nucleotide polymorphism-based visual identification of *Rhodiola crenulata* using loop-mediated isothermal amplification technique

Li Hao[1], Xin Shi[1], Shiyu Wen[1], Caiye Yang[1], Yaqi Chen[1], Samo Yue[1], Jiaqiang Chen[1], Kexin Luo[1], Bingliang Liu[1], Yanxia Sun[1,*], Yi Zhang[2,*]

*** Correspondence:**

Yanxia Sun

sunyanxia1976@cdu.edu.cn

Yi Zhang

zhangyi1@cib.ac.cn

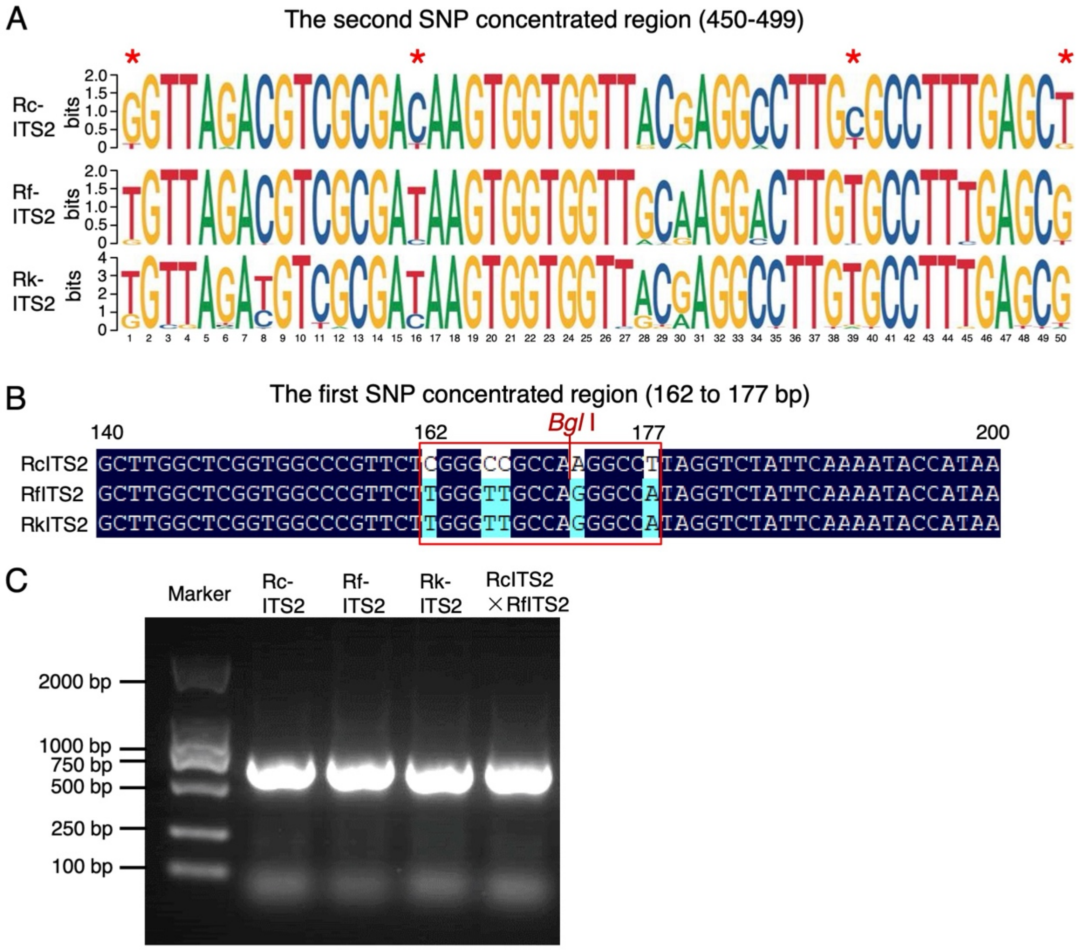

Supplementary Fig. 1 Sequence analysis of ITS2 fragments from *Rhodiola* species. (A) Base preference analysis within the second SNP-concentrated region (450 to 499 bp). Potential SNP sites are highlighted with red stars. **(B)** Multiple alignment of the sequenced ITS2 fragments from three *Rhodiola* species. The red box indicates the first SNP-concentrated region (162 to 177 bp). **(C)** Agarose gel electrophoresis of ITS2 amplification products from four *Rhodiola* species. Marker, DL2000 DNA ladder.

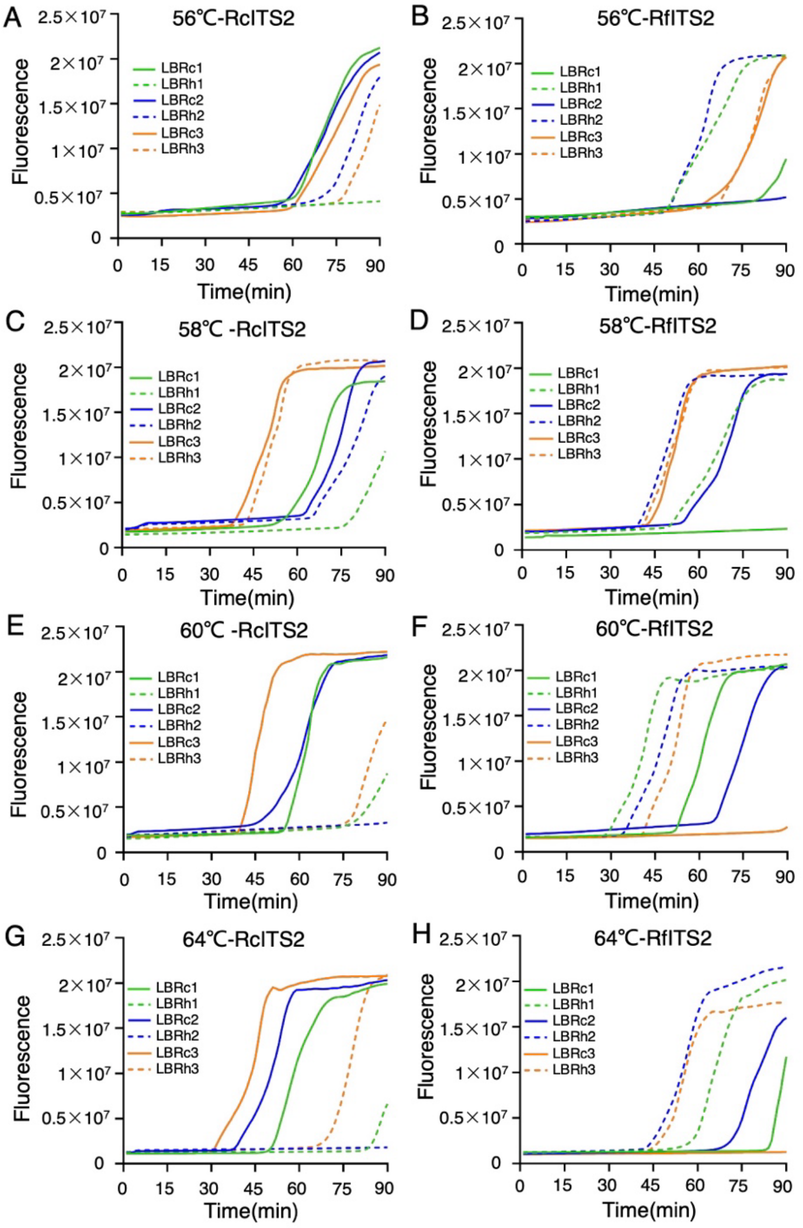

Supplementary Fig. 2 Discrimination plot of real-time fluorescence LAMP reaction under varying temperature gradients. **(A), (C), (E), (G)** Discrimination plot of real-time fluorescence LAMP reactions using LBRc primers at temperatures of 56°C, 58°C, 60°C, and 64°C. **(B), D), (F), (H)** Discrimination plot of real-time fluorescence LAMP reactions using LBRh primers at the same temperature settings.

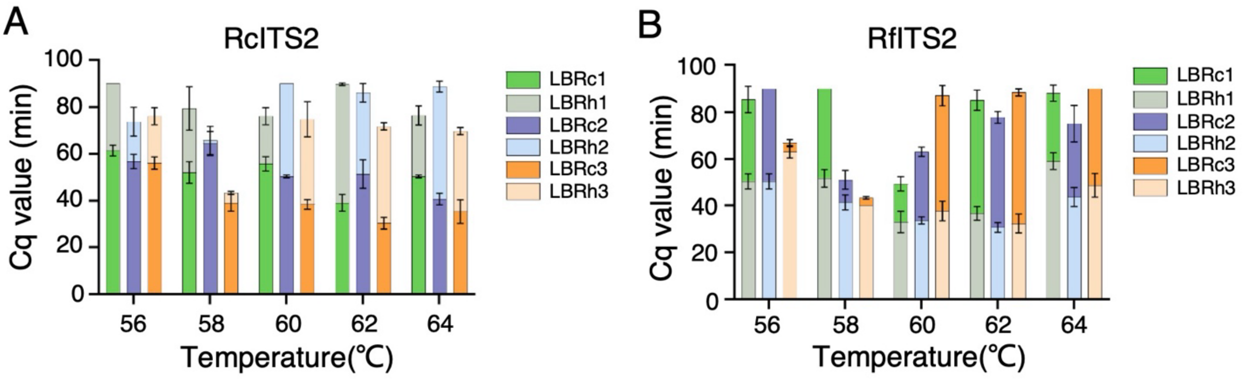

Supplementary Fig. 3 Time gaps of *Rhodiola* species detection using specific LB primers under temperature gradients. (A) The detection time (Cq) of specific LB primers using RcITS2 plasmid templates under 56℃, 58℃, 60℃, 62℃, and 64℃. (**B)** The detection time (Cq) of specific LB primers using RhITS2 plasmid templates under 56℃, 58℃, 60℃, 62℃, and 64℃. Error bars represent mean ± standard deviation (SD).

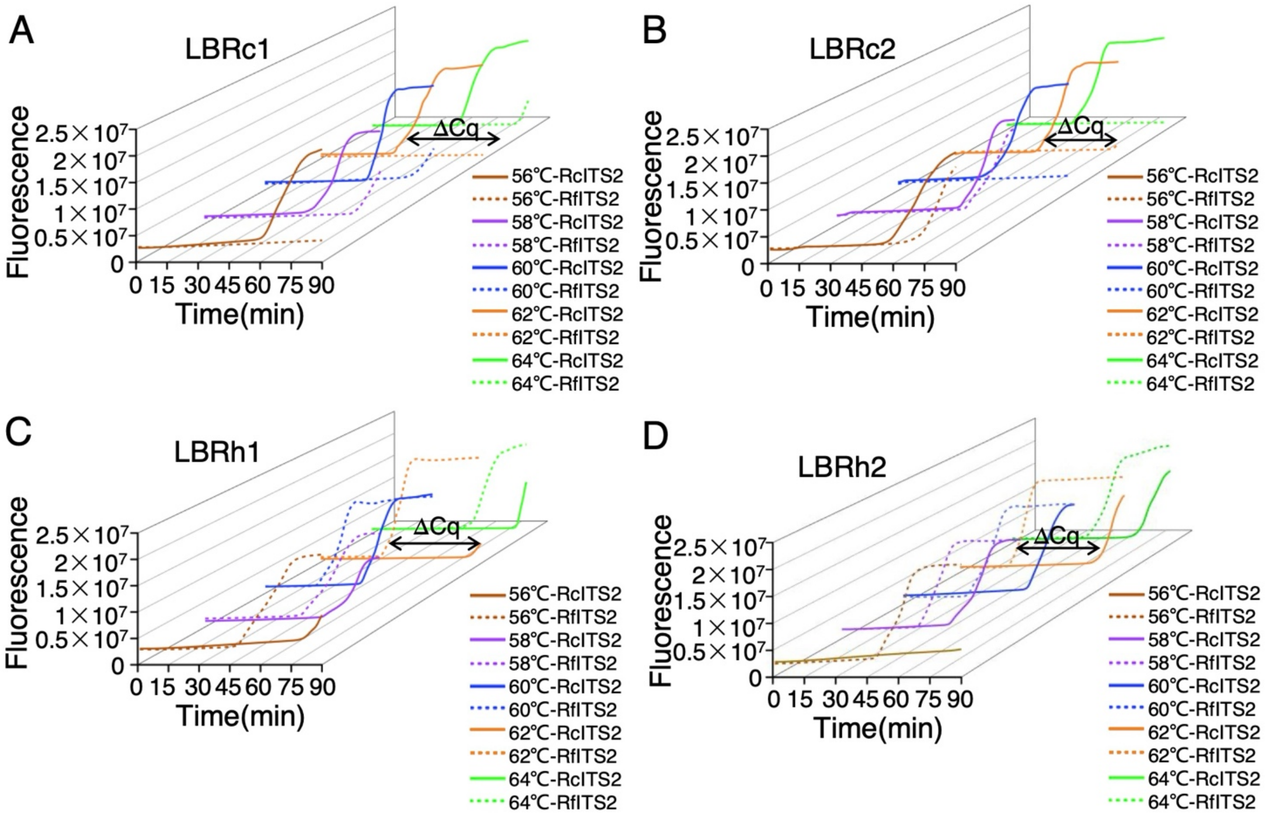

Supplementary Fig. 4 Three dimensional (3D) view of the time gap between RcITS2 and RfITS2 detection using specific LB primers under temperature gradients. **(A)-(B)** The time gap (ΔCq) between RcITS2 and RfITS2 detection using LBRc1 (**A**) and LBRc2 (**B**) under 56°C, 58°C, 60°C, 62°C, and 64°C in 3D view. **(C)-(D)** The time gap (ΔCq) between RcITS2 and RfITS2 detection using LBRh1 (**C**) and LBRh2 (**D**) under 56°C, 58°C, 60°C, 62°C, and 64°C in 3D view.

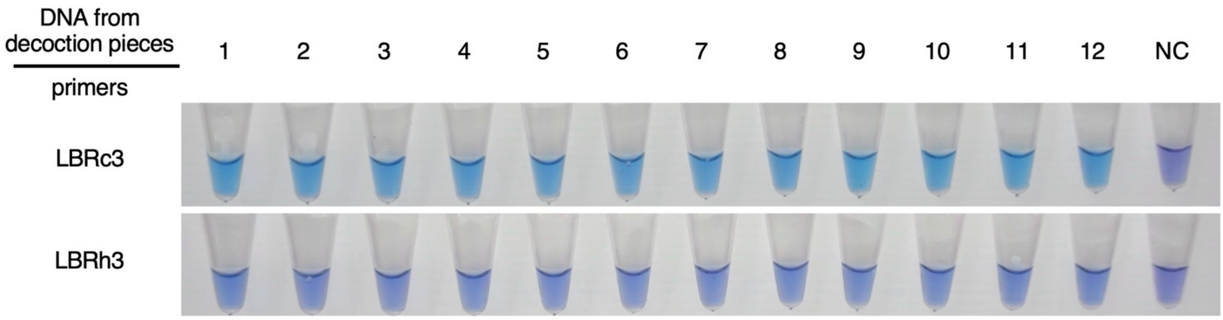

Supplementary Fig. 5 Application of visual LAMP system with HNB in *Rhodiola* decoction pieces identification. NC, negative control. Each experiment was repeated three times.

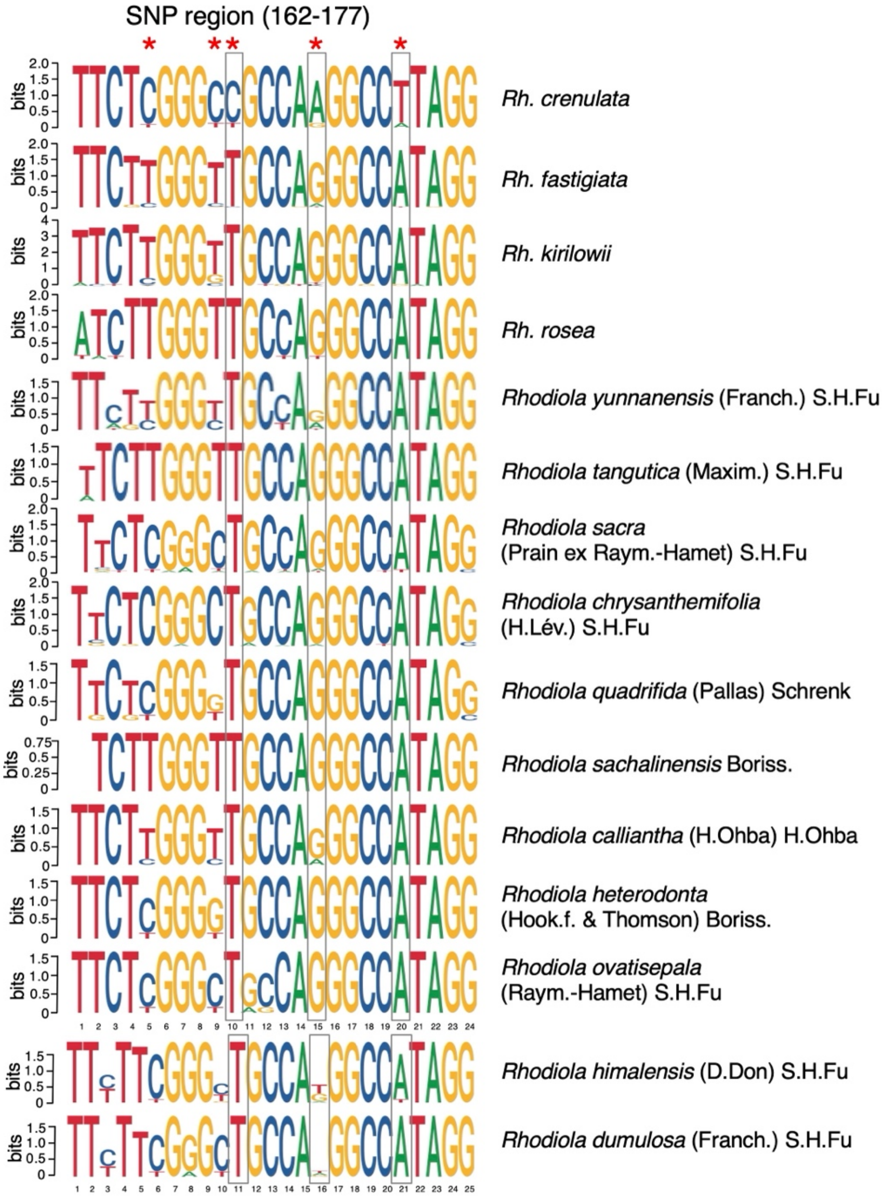

Supplementary Fig. 6 Base preference analysis of the first SNP-concentrated region (162 to 177 bp) in ITS2 from *Rhodiola* species. The potential SNP sites are highlighted with red stars.
