# Supplementary material for: Single nucleotide polymorphism-based visual identification of Rhodiola crenulata using the loop-mediated isothermal amplification technique
Source: Front Plant Sci. 2025 Jan 16;15:1492083. doi: 10.3389/fpls.2024.1492083 (PMC11779703; doi:10.3389/fpls.2024.1492083)
Supplement: Supplementary file 2 [file Table1.docx]

Supplementary tables

Single nucleotide polymorphism (SNP)-based visual identification of *Rhodiola* *crenulata* using loop-mediated isothermal amplification (LAMP) technique

Li Hao^1^, Xin Shi^1^, Shiyu Wen^1^, Caiye Yang^1^, Yaqi Chen^1^, Samo Yue^1^, Jiaqiang Chen^1^, Kexin Luo^1^, Bingliang Liu^1^, Yanxia Sun^1,^*, Yi Zhang^2,^*

*** Correspondence:**

Yanxia Sun

sunyanxia1976@cdu.edu.cn

Yi Zhang

zhangyi1@cib.ac.cn

# Supplementary table 1. Plant sample information.

| **Latin name** | **Location** | **Collection date** | **Collection site** | **Provider** | **Identifier** | **Voucher specimen** |
| --- | --- | --- | --- | --- | --- | --- |
| *Rhodiola crenulata* (J. D. Hooker & Thomson) H. Ohba | Xizang | 2023/04/25 | Leaves | Xizang Rhodiola Pharmaceutical Holding Company, Xizang, China | Yuehua Wang | Y.S. Chen et al. 13-0349 (PE) |
| *Rhodiola kirilowii* (Regel) Maximowicz | Xizang | 2023/04/25 | Leaves | Xizang Rhodiola Pharmaceutical Holding Company, Xizang, China | Yuehua Wang | FLPH Xizang Expedition 12-0465 (PE) |
| *Rhodiola fastigiata* (J. D. Hooker & Thomson) S. H. Fu | Xizang | 2023/04/25 | Leaves | Xizang Rhodiola Pharmaceutical Holding Company, Xizang, China | Yuehua Wang | PE Xizang Expedition PE6468 (PE) |
| *R. crenulata* × *R. fastigiata* | Xizang | 2023/04/25 | Leaves | Xizang Rhodiola Pharmaceutical Holding Company, Xizang, China | Yuehua Wang | - |

| **Supplementary table 2. Accession numbers of *Rhodiola* ITS2 sequences in GenBank.** | |
| --- | --- |
| **Taxon** | **ITS of GenBank accession number** |
| *Rhodiola crenulata* (J. D. Hooker & Thomson) H. Ohba | MH371168.1 |
|  | MH371167.1 |
|  | MH371166.1 |
|  | MH371165.1 |
|  | MH371164.1 |
|  | MH371163.1 |
|  | KF113691.1 |
|  | JF978175.1 |
|  | JF978174.1 |
|  | OL818325.1 |
|  | OL818321.1 |
|  | MG917778.1 |
|  | KM211543.1 |
|  | KP114718.1 |
|  | KJ569927.1 |
|  | KJ569926.1 |
|  | EU239667.1 |
|  | EU239666.1 |
|  | EU239665.1 |
|  | AY359897.1 |
|  | AY359896.1 |
|  | AY359895.1 |
|  | AY359894.1 |
|  | AY359893.1 |
|  | AY359892.1 |
|  | AY359891.1 |
|  | AY359890.1 |
|  | AY359889.1 |
|  | AY359888.1 |
|  | AY352898.1 |
|  | MH258175.1 |
|  | MH258174.1 |
|  | MH258173.1 |
|  | MH258172.1 |
|  | KT285136.1 |
|  | KT285135.1 |
|  | KT285134.1 |
|  | KT365799.1 |
|  | KJ796856.1 |
|  | KJ796855.1 |
|  | GQ434463.1 |
|  | OK666428.1 |
|  | OK666423.1 |
| *Rhodiola fastigiata* (J. D. Hooker & Thomson) S. H. Fu | MH023237.1 |
|  | MH023236.1 |
|  | MH023235.1 |
|  | MH023234.1 |
|  | MH023233.1 |
|  | MH023232.1 |
|  | MH023231.1 |
|  | MH023230.1 |
|  | MH023229.1 |
|  | MH023228.1 |
|  | MH023227.1 |
|  | MH023226.1 |
|  | MH023225.1 |
|  | MH023224.1 |
|  | MH023223.1 |
|  | MH023222.1 |
|  | MH023221.1 |
|  | MH023220.1 |
|  | MH023219.1 |
|  | KF113695.1 |
|  | JF978177.1 |
|  | JF978176.1 |
|  | OL818320.1 |
|  | KY936942.1 |
|  | KY936941.1 |
|  | KY936940.1 |
|  | KY936939.1 |
|  | KY936938.1 |
|  | KY936937.1 |
|  | KY936936.1 |
|  | KY936935.1 |
|  | KY936934.1 |
|  | KY936933.1 |
|  | KY936932.1 |
|  | KY936931.1 |
|  | KY936930.1 |
|  | KP114728.1 |
|  | KP114727.1 |
|  | KJ569932.1 |
|  | KJ569931.1 |
|  | KJ569930.1 |
|  | KJ569929.1 |
|  | EU239672.1 |
|  | AY359906.1 |
|  | AY359905.1 |
|  | AY359904.1 |
|  | AY359903.1 |
|  | AY359902.1 |
|  | AY359901.1 |
|  | AY359900.1 |
|  | AY359899.1 |
|  | AY359898.1 |
|  | AB088594.1 |
|  | KJ796858.1 |
|  | KJ796857.1 |
|  | OK666425.1 |
|  | OK666417.1 |
|  | OK666414.1 |
| *Rhodiola kirilowii* (Regel) Maximowicz | GQ374206.1 |
|  | KM459563.1 |
|  | KM459562.1 |
|  | KM459561.1 |
|  | KM459560.1 |
|  | KM459559.1 |
|  | KM459558.1 |
|  | KM459557.1 |
|  | KM459556.1 |
|  | KM459555.1 |
|  | KM459554.1 |
|  | KM459553.1 |
|  | KM459552.1 |
|  | KF113705.1 |
|  | JF978180.1 |
|  | JF978179.1 |
|  | JF978178.1 |
|  | MT924101.1 |
|  | MT924100.1 |
|  | MT924099.1 |
|  | OP673520.1 |
|  | MH712654.1 |
|  | MH711393.1 |
|  | KP114749.1 |
|  | KP114748.1 |
|  | KP114747.1 |
|  | KP114746.1 |
|  | KJ569941.1 |
|  | EU239669.1 |
|  | EU239668.1 |
|  | AB088601.1 |
|  | KY767815.1 |
|  | KU508331.1 |
|  | KF895362.1 |
|  | KF895361.1 |
|  | KF895360.1 |
|  | KF895359.1 |
|  | KF895358.1 |
|  | KF895357.1 |
|  | KF895356.1 |
|  | KF454613.1 |
|  | KF454612.1 |
|  | KF454611.1 |
|  | KF454610.1 |
|  | KF454609.1 |
|  | KF454608.1 |
|  | KJ796861.1 |
|  | OK666413.1 |

| **Supplementary table 3. Primers used in this study.** | |
| --- | --- |
| **Primer name** | **Sequence (5'-3')** |
| RhITS1-F | TCCGTAGGTGAACCTGCGG |
| RhITS4-R | TCCTCCGCTTATTGATATGC |
| F3 | TGGATGCACTTGGATCCTTC |
| B3 | CGAGAGCCGAGATATCCGT |
| F1C-F2 | TTCAATCAGCGATGGGCGCAGGGTACTCCATTTGCCTTCC |
| B1C-B2 | GAAGAGCTTGGCTCGGTGGCTGCCGAGAGTCGTTATGGTA |
| LBRc1 | **C**GGG**CC**GCCA**A** |
| LBRh1 | **T**GGG**TT**GCCA**G** |
| LBRc2 | **C**GCCA**A**GGCC**T** |
| LBRh2 | **T**GCCA**G**GGCC**A** |
| LBRc3 | **CC**GCCA**A**GGCC**T**TAGGT |
| LBRh3 | **TT**GCCA**G**GGCC**A**TAGGT |
|  |  |
| The SNP sites were highlighted with bold. | |

| **Supplementary table 4. Intraspecific and interspecific Pi values of *Rhodiola* ITS2.** | | |
| --- | --- | --- |
| **position** | **Rc_pi** | **interspecific_pi** |
| 1 | 0 | 0.01754 |
| 2 | 0 | 0.01754 |
| 3 | 0 | 0.01754 |
| 4 | 0 | 0.01754 |
| 5 | 0 | 0.01754 |
| 6 | 0 | 0.01754 |
| 7 | 0 | 0.01754 |
| 8 | 0 | 0.01754 |
| 9 | 0 | 0.01754 |
| 10 | 0 | 0.01754 |
| 11 | 0 | 0.01754 |
| 12 | 0 | 0.01754 |
| 13 | 0 | 0.01754 |
| 14 | 0 | 0.01754 |
| 15 | 0 | 0.01754 |
| 16 | 0 | 0.01754 |
| 17 | 0 | 0.01754 |
| 18 | 0.18621 | 0.01754 |
| 19 | 0 | 0.01754 |
| 20 | 0 | 0.01754 |
| 21 | 0 | 0.01754 |
| 22 | 0 | 0.01754 |
| 23 | 0 | 0.01754 |
| 24 | 0 | 0.01754 |
| 25 | 0 | 0.01754 |
| 26 | 0 | 0.01754 |
| 27 | 0 | 0.01754 |
| 28 | 0 | 0.01754 |
| 29 | 0 | 0.01754 |
| 30 | 0 | 0.01754 |
| 31 | 0 | 0.01754 |
| 32 | 0 | 0.01754 |
| 33 | 0 | 0.01754 |
| 34 | 0.06667 | 0.01754 |
| 35 | 0.06667 | 0.01754 |
| 36 | 0.06667 | 0.01754 |
| 37 | 0.06667 | 0.01754 |
| 38 | 0.06667 | 0.01754 |
| 39 | 0.06667 | 0.01754 |
| 40 | 0.06667 | 0.01754 |
| 41 | 0 | 0.01754 |
| 42 | 0.06667 | 0.01754 |
| 43 | 0 | 0.01754 |
| 44 | 0 | 0.01754 |
| 45 | 0 | 0.01754 |
| 46 | 0 | 0.01754 |
| 47 | 0 | 0.01754 |
| 48 | 0 | 0.01754 |
| 49 | 0 | 0.01754 |
| 50 | 0 | 0.01754 |
| 51 | 0 | 0.01754 |
| 52 | 0.18621 | 0.01754 |
| 53 | 0 | 0.01754 |
| 54 | 0 | 0.01754 |
| 55 | 0 | 0.01754 |
| 56 | 0 | 0.01754 |
| 57 | 0 | 0.01754 |
| 58 | 0 | 0.01754 |
| 59 | 0 | 0.01754 |
| 60 | 0 | 0.01754 |
| 61 | 0 | 0.01754 |
| 62 | 0 | 0.01754 |
| 63 | 0 | 0.01754 |
| 64 | 0 | 0.01754 |
| 65 | 0 | 0.01754 |
| 66 | 0 | 0.01754 |
| 67 | 0 | 0.01754 |
| 68 | 0 | 0.01754 |
| 69 | 0 | 0.01754 |
| 70 | 0 | 0 |
| 71 | 0 | 0 |
| 72 | 0 | 0 |
| 73 | 0 | 0.01754 |
| 74 | 0 | 0.06831 |
| 75 | 0 | 0 |
| 76 | 0 | 0.33163 |
| 77 | 0.06667 | 0.03493 |
| 78 | 0 | 0.01754 |
| 79 | 0 | 0.06909 |
| 80 | 0 | 0.01754 |
| 81 | 0.18621 | 0.47136 |
| 82 | 0 | 0 |
| 83 | 0 | 0.03478 |
| 84 | 0 | 0.08461 |
| 85 | 0 | 0 |
| 86 | 0 | 0.01754 |
| 87 | 0 | 0.03493 |
| 88 | 0.18621 | 0.3827 |
| 89 | 0 | 0 |
| 90 | 0 | 0 |
| 91 | 0 | 0 |
| 92 | 0 | 0.03493 |
| 93 | 0 | 0.01754 |
| 94 | 0 | 0.01754 |
| 95 | 0 | 0 |
| 96 | 0 | 0 |
| 97 | 0 | 0.01754 |
| 98 | 0 | 0 |
| 99 | 0 | 0 |
| 100 | 0 | 0 |
| 101 | 0 | 0.01754 |
| 102 | 0 | 0 |
| 103 | 0 | 0 |
| 104 | 0 | 0 |
| 105 | 0 | 0 |
| 106 | 0 | 0 |
| 107 | 0 | 0 |
| 108 | 0 | 0 |
| 109 | 0 | 0 |
| 110 | 0 | 0 |
| 111 | 0 | 0.01754 |
| 112 | 0 | 0.01754 |
| 113 | 0 | 0.03478 |
| 114 | 0 | 0.35259 |
| 115 | 0 | 0 |
| 116 | 0 | 0 |
| 117 | 0 | 0 |
| 118 | 0 | 0 |
| 119 | 0 | 0 |
| 120 | 0 | 0 |
| 121 | 0 | 0.01754 |
| 122 | 0 | 0 |
| 123 | 0 | 0 |
| 124 | 0 | 0 |
| 125 | 0 | 0 |
| 126 | 0 | 0 |
| 127 | 0 | 0 |
| 128 | 0.18621 | 0.45412 |
| 129 | 0 | 0.01754 |
| 130 | 0 | 0.01754 |
| 131 | 0 | 0 |
| 132 | 0.12874 | 0.11815 |
| 133 | 0 | 0.01754 |
| 134 | 0 | 0 |
| 135 | 0.18621 | 0.3827 |
| 136 | 0 | 0 |
| 137 | 0 | 0.03493 |
| 138 | 0 | 0 |
| 139 | 0 | 0.0517 |
| 140 | 0 | 0.0517 |
| 141 | 0 | 0 |
| 142 | 0 | 0.03493 |
| 143 | 0 | 0.03478 |
| 144 | 0.06667 | 0.03478 |
| 145 | 0 | 0.01754 |
| 146 | 0 | 0.01754 |
| 147 | 0 | 0.03478 |
| 148 | 0 | 0.08555 |
| 149 | 0 | 0.03478 |
| 150 | 0 | 0 |
| 151 | 0.13103 | 0.05201 |
| 152 | 0 | 0 |
| 153 | 0 | 0 |
| 154 | 0 | 0.01754 |
| 155 | 0 | 0 |
| 156 | 0 | 0 |
| 157 | 0 | 0.03478 |
| 158 | 0 | 0.03478 |
| 159 | 0 | 0.01754 |
| 160 | 0 | 0.01754 |
| 161 | 0 | 0.0517 |
| 162 | 0.12874 | 0.45412 |
| 163 | 0 | 0 |
| 164 | 0 | 0 |
| 165 | 0 | 0 |
| 166 | 0.18621 | 0.49542 |
| 167 | 0.18621 | 0.37385 |
| 168 | 0 | 0 |
| 169 | 0 | 0.01754 |
| 170 | 0 | 0.01754 |
| 171 | 0 | 0.03493 |
| 172 | 0.18621 | 0.43704 |
| 173 | 0 | 0 |
| 174 | 0 | 0 |
| 175 | 0 | 0.01754 |
| 176 | 0 | 0 |
| 177 | 0.18621 | 0.38705 |
| 178 | 0 | 0.01754 |
| 179 | 0 | 0.01754 |
| 180 | 0 | 0 |
| 181 | 0 | 0 |
| 182 | 0 | 0 |
| 183 | 0 | 0 |
| 184 | 0 | 0 |
| 185 | 0 | 0 |
| 186 | 0 | 0 |
| 187 | 0 | 0.01754 |
| 188 | 0 | 0.01754 |
| 189 | 0 | 0 |
| 190 | 0 | 0 |
| 191 | 0 | 0 |
| 192 | 0 | 0 |
| 193 | 0 | 0 |
| 194 | 0 | 0 |
| 195 | 0 | 0.01754 |
| 196 | 0 | 0.03493 |
| 197 | 0 | 0 |
| 198 | 0 | 0.06831 |
| 199 | 0 | 0 |
| 200 | 0 | 0 |
| 201 | 0 | 0.01754 |
| 202 | 0 | 0 |
| 203 | 0 | 0 |
| 204 | 0 | 0 |
| 205 | 0 | 0 |
| 206 | 0 | 0 |
| 207 | 0 | 0 |
| 208 | 0 | 0 |
| 209 | 0 | 0 |
| 210 | 0 | 0 |
| 211 | 0 | 0 |
| 212 | 0 | 0 |
| 213 | 0 | 0 |
| 214 | 0 | 0 |
| 215 | 0 | 0 |
| 216 | 0 | 0 |
| 217 | 0 | 0 |
| 218 | 0 | 0 |
| 219 | 0 | 0 |
| 220 | 0 | 0 |
| 221 | 0 | 0 |
| 222 | 0 | 0 |
| 223 | 0 | 0 |
| 224 | 0 | 0 |
| 225 | 0 | 0 |
| 226 | 0 | 0 |
| 227 | 0 | 0 |
| 228 | 0 | 0 |
| 229 | 0 | 0 |
| 230 | 0 | 0 |
| 231 | 0 | 0 |
| 232 | 0 | 0 |
| 233 | 0 | 0 |
| 234 | 0 | 0 |
| 235 | 0 | 0 |
| 236 | 0 | 0 |
| 237 | 0 | 0 |
| 238 | 0 | 0 |
| 239 | 0 | 0 |
| 240 | 0 | 0 |
| 241 | 0 | 0 |
| 242 | 0 | 0.0517 |
| 243 | 0 | 0 |
| 244 | 0 | 0 |
| 245 | 0 | 0 |
| 246 | 0 | 0 |
| 247 | 0 | 0 |
| 248 | 0 | 0 |
| 249 | 0 | 0.01754 |
| 250 | 0 | 0 |
| 251 | 0 | 0.01754 |
| 252 | 0 | 0 |
| 253 | 0 | 0 |
| 254 | 0 | 0 |
| 255 | 0 | 0 |
| 256 | 0 | 0 |
| 257 | 0 | 0 |
| 258 | 0 | 0 |
| 259 | 0 | 0 |
| 260 | 0 | 0 |
| 261 | 0 | 0 |
| 262 | 0 | 0 |
| 263 | 0 | 0 |
| 264 | 0 | 0 |
| 265 | 0 | 0 |
| 266 | 0 | 0 |
| 267 | 0 | 0 |
| 268 | 0 | 0 |
| 269 | 0 | 0 |
| 270 | 0 | 0 |
| 271 | 0 | 0 |
| 272 | 0 | 0 |
| 273 | 0 | 0 |
| 274 | 0 | 0 |
| 275 | 0 | 0 |
| 276 | 0 | 0 |
| 277 | 0 | 0 |
| 278 | 0 | 0.01754 |
| 279 | 0 | 0 |
| 280 | 0 | 0 |
| 281 | 0 | 0 |
| 282 | 0 | 0 |
| 283 | 0 | 0 |
| 284 | 0 | 0 |
| 285 | 0 | 0 |
| 286 | 0 | 0 |
| 287 | 0 | 0 |
| 288 | 0 | 0 |
| 289 | 0 | 0 |
| 290 | 0 | 0 |
| 291 | 0 | 0 |
| 292 | 0 | 0 |
| 293 | 0 | 0 |
| 294 | 0 | 0 |
| 295 | 0 | 0 |
| 296 | 0 | 0 |
| 297 | 0 | 0 |
| 298 | 0 | 0 |
| 299 | 0 | 0.01754 |
| 300 | 0 | 0 |
| 301 | 0 | 0 |
| 302 | 0 | 0 |
| 303 | 0 | 0 |
| 304 | 0 | 0 |
| 305 | 0 | 0 |
| 306 | 0 | 0 |
| 307 | 0 | 0 |
| 308 | 0 | 0 |
| 309 | 0 | 0 |
| 310 | 0 | 0 |
| 311 | 0 | 0 |
| 312 | 0 | 0 |
| 313 | 0 | 0 |
| 314 | 0 | 0 |
| 315 | 0 | 0 |
| 316 | 0 | 0 |
| 317 | 0 | 0 |
| 318 | 0 | 0 |
| 319 | 0 | 0 |
| 320 | 0 | 0.06831 |
| 321 | 0 | 0 |
| 322 | 0 | 0 |
| 323 | 0 | 0 |
| 324 | 0 | 0.01754 |
| 325 | 0 | 0 |
| 326 | 0 | 0 |
| 327 | 0 | 0.01754 |
| 328 | 0 | 0 |
| 329 | 0 | 0 |
| 330 | 0 | 0 |
| 331 | 0 | 0 |
| 332 | 0 | 0 |
| 333 | 0 | 0.3175 |
| 334 | 0 | 0.03478 |
| 335 | 0 | 0 |
| 336 | 0 | 0 |
| 337 | 0 | 0 |
| 338 | 0 | 0 |
| 339 | 0 | 0 |
| 340 | 0 | 0 |
| 341 | 0 | 0 |
| 342 | 0 | 0 |
| 343 | 0 | 0 |
| 344 | 0 | 0 |
| 345 | 0 | 0 |
| 346 | 0 | 0 |
| 347 | 0 | 0 |
| 348 | 0 | 0 |
| 349 | 0 | 0 |
| 350 | 0 | 0 |
| 351 | 0 | 0 |
| 352 | 0 | 0 |
| 353 | 0 | 0.01754 |
| 354 | 0 | 0 |
| 355 | 0 | 0 |
| 356 | 0 | 0 |
| 357 | 0 | 0 |
| 358 | 0 | 0 |
| 359 | 0.18621 | 0.45956 |
| 360 | 0 | 0 |
| 361 | 0 | 0 |
| 362 | 0 | 0 |
| 363 | 0.18621 | 0.37385 |
| 364 | 0 | 0 |
| 365 | 0 | 0 |
| 366 | 0 | 0 |
| 367 | 0 | 0 |
| 368 | 0 | 0.01754 |
| 369 | 0 | 0.05201 |
| 370 | 0 | 0 |
| 371 | 0 | 0 |
| 372 | 0 | 0 |
| 373 | 0 | 0 |
| 374 | 0 | 0 |
| 375 | 0 | 0.01754 |
| 376 | 0 | 0 |
| 377 | 0 | 0 |
| 378 | 0 | 0 |
| 379 | 0 | 0 |
| 380 | 0 | 0 |
| 381 | 0 | 0 |
| 382 | 0 | 0.03478 |
| 383 | 0 | 0.01754 |
| 384 | 0 | 0.01754 |
| 385 | 0 | 0.03478 |
| 386 | 0 | 0 |
| 387 | 0 | 0 |
| 388 | 0 | 0 |
| 389 | 0 | 0.03478 |
| 390 | 0 | 0 |
| 391 | 0 | 0.03478 |
| 392 | 0 | 0 |
| 393 | 0 | 0 |
| 394 | 0 | 0 |
| 395 | 0 | 0 |
| 396 | 0 | 0 |
| 397 | 0 | 0 |
| 398 | 0 | 0 |
| 399 | 0 | 0 |
| 400 | 0 | 0 |
| 401 | 0 | 0 |
| 402 | 0 | 0 |
| 403 | 0 | 0 |
| 404 | 0.18621 | 0.50054 |
| 405 | 0 | 0 |
| 406 | 0 | 0 |
| 407 | 0 | 0 |
| 408 | 0 | 0 |
| 409 | 0 | 0 |
| 410 | 0 | 0.01754 |
| 411 | 0 | 0.01754 |
| 412 | 0 | 0.01754 |
| 413 | 0 | 0.03478 |
| 414 | 0 | 0 |
| 415 | 0 | 0.06878 |
| 416 | 0 | 0.01754 |
| 417 | 0 | 0 |
| 418 | 0 | 0.01754 |
| 419 | 0 | 0 |
| 420 | 0 | 0 |
| 421 | 0 | 0 |
| 422 | 0 | 0 |
| 423 | 0 | 0 |
| 424 | 0 | 0 |
| 425 | 0 | 0 |
| 426 | 0 | 0 |
| 427 | 0 | 0 |
| 428 | 0 | 0 |
| 429 | 0 | 0 |
| 430 | 0 | 0 |
| 431 | 0 | 0 |
| 432 | 0 | 0 |
| 433 | 0 | 0 |
| 434 | 0 | 0 |
| 435 | 0 | 0 |
| 436 | 0 | 0 |
| 437 | 0 | 0 |
| 438 | 0 | 0 |
| 439 | 0 | 0 |
| 440 | 0 | 0 |
| 441 | 0 | 0 |
| 442 | 0 | 0 |
| 443 | 0 | 0.01754 |
| 444 | 0 | 0 |
| 445 | 0 | 0 |
| 446 | 0 | 0.01754 |
| 447 | 0 | 0 |
| 448 | 0 | 0 |
| 449 | 0 | 0 |
| 450 | 0.18621 | 0.45956 |
| 451 | 0 | 0 |
| 452 | 0 | 0.03478 |
| 453 | 0 | 0.03478 |
| 454 | 0 | 0 |
| 455 | 0.06667 | 0.06909 |
| 456 | 0 | 0 |
| 457 | 0 | 0.3175 |
| 458 | 0 | 0 |
| 459 | 0 | 0 |
| 460 | 0 | 0.0517 |
| 461 | 0 | 0.01754 |
| 462 | 0 | 0 |
| 463 | 0 | 0 |
| 464 | 0 | 0 |
| 465 | 0.18621 | 0.45956 |
| 466 | 0 | 0 |
| 467 | 0 | 0 |
| 468 | 0 | 0 |
| 469 | 0 | 0 |
| 470 | 0 | 0 |
| 471 | 0 | 0 |
| 472 | 0 | 0 |
| 473 | 0 | 0 |
| 474 | 0 | 0 |
| 475 | 0 | 0 |
| 476 | 0 | 0.01754 |
| 477 | 0.12874 | 0.50442 |
| 478 | 0 | 0.06909 |
| 479 | 0.18621 | 0.50442 |
| 480 | 0 | 0 |
| 481 | 0 | 0 |
| 482 | 0 | 0 |
| 483 | 0.12874 | 0.49449 |
| 484 | 0 | 0.01754 |
| 485 | 0 | 0 |
| 486 | 0 | 0 |
| 487 | 0 | 0.01754 |
| 488 | 0.4046 | 0.35274 |
| 489 | 0 | 0.01754 |
| 490 | 0 | 0 |
| 491 | 0 | 0 |
| 492 | 0 | 0 |
| 493 | 0 | 0 |
| 494 | 0 | 0.06878 |
| 495 | 0 | 0 |
| 496 | 0 | 0 |
| 497 | 0 | 0.01754 |
| 498 | 0 | 0.01754 |
| 499 | 0.18621 | 0.42742 |
| 500 | 0 | 0 |
| 501 | 0 | 0 |
| 502 | 0 | 0 |
| 503 | 0 | 0 |
| 504 | 0 | 0.06831 |
| 505 | 0.18621 | 0.38705 |
| 506 | 0 | 0 |
| 507 | 0 | 0 |
| 508 | 0 | 0.06878 |
| 509 | 0 | 0 |
| 510 | 0 | 0 |
| 511 | 0 | 0 |
| 512 | 0 | 0 |
| 513 | 0 | 0 |
| 514 | 0 | 0 |
| 515 | 0 | 0 |
| 516 | 0 | 0 |
| 517 | 0 | 0.01754 |
| 518 | 0 | 0 |
| 519 | 0 | 0.08461 |
| 520 | 0 | 0.01754 |
| 521 | 0 | 0.01754 |
| 522 | 0 | 0.21938 |
| 523 | 0 | 0.01754 |
| 524 | 0 | 0 |
| 525 | 0 | 0.03493 |
| 526 | 0 | 0.03493 |
| 527 | 0 | 0.03493 |
| 528 | 0 | 0.05201 |
| 529 | 0 | 0.01754 |
| 530 | 0 | 0.01754 |
| 531 | 0 | 0.01754 |
| 532 | 0 | 0 |
| 533 | 0 | 0 |
| 534 | 0 | 0 |
| 535 | 0 | 0.03493 |
| 536 | 0 | 0.05201 |
| 537 | 0 | 0.01754 |
| 538 | 0 | 0 |
| 539 | 0 | 0 |
| 540 | 0 | 0 |
| 541 | 0 | 0 |
| 542 | 0 | 0 |
| 543 | 0 | 0 |
| 544 | 0 | 0 |
| 545 | 0 | 0.03478 |
| 546 | 0 | 0 |
| 547 | 0 | 0 |
| 548 | 0.18621 | 0 |
| 549 | 0 | 0 |
| 550 | 0 | 0.01754 |
| 551 | 0 | 0.01754 |
| 552 | 0 | 0.01754 |
| 553 | 0 | 0 |

| **Supplementary table 5. Statistical analysis of the detection time of the specific LB primers under temperature gradients.** | | | | |
| --- | --- | --- | --- | --- |
| **Primer name** | **Temperature (℃)** | **Mean Cq value ± standard deviation (min)** | **Significance analysis**  **(*P* < 0.05)** | **Significance analysis**  **(*P* < 0.01)** |
| LBRc1 | 56 | 61.33±2.31 | c | C |
|  | 58 | 52.00±4.58 | b | B |
|  | 60 | 55.67±3.06 | bc | BC |
|  | 62 | 39.00±3.61 | a | A |
|  | 64 | 50.33±0.58 | b | B |
| LBRc2 | 56 | 56.67±3.06 | b | BC |
|  | 58 | 64.33±5.13 | c | C |
|  | 60 | 50.33±0.58 | b | AB |
|  | 62 | 51.33±6.11 | b | AB |
|  | 64 | 40.67±2.52 | a | A |
| LBRc3 | 56 | 56.00±2.65 | c | B |
|  | 58 | 39.00±3.61 | b | A |
|  | 60 | 38.33±2.08 | b | A |
|  | 62 | 30.33±2.52 | a | A |
|  | 64 | 35.33±5.03 | ab | A |
| LBRh1 | 56 | 50.33±3.21 | b | B |
|  | 58 | 51.67±3.79 | b | B |
|  | 60 | 33.00±4.58 | a | A |
|  | 62 | 36.67±2.89 | a | A |
|  | 64 | 59.00±3.61 | c | B |
| LBRh2 | 56 | 50.33±3.21 | c | C |
|  | 58 | 41.33±3.21 | b | B |
|  | 60 | 33.67±1.53 | a | A |
|  | 62 | 30.67±2.08 | a | A |
|  | 64 | 43.67±4.04 | b | BC |
| LBRh3 | 56 | 63.00±2.65 | c | C |
|  | 58 | 40.00±0.00 | b | AB |
|  | 60 | 37.67±4.16 | ab | A |
|  | 62 | 32.33±4.04 | a | A |
|  | 64 | 48.67±5.03 | c | B |
